# Differential impacts of the COVID-19 pandemic on sociodemographic groups: A mathematical model framework

**DOI:** 10.1371/journal.pone.0330273

**Published:** 2026-01-27

**Authors:** Gbeminiyi J. Oyedele, Ivo Vlaev, Michael J. Tildesley

**Affiliations:** 1 Warwick Medical School/Institute for Global and Pandemic Planning, University of Warwick, Coventry, United Kingdom; 2 Centre for Behavioural and Implementation Science Intervention (BISI), Yong Loo Lin School of Medicine, National University of Singapore, Singapore, Singapore; 3 School of Life Science and Mathematics, University of Warwick, Coventry, United Kingdom; United Arab Emirates University Faculty of Science: UAE University College of Science, UNITED ARAB EMIRATES

## Abstract

Deprivation and age can both drive disparities in infectious disease transmission and outcomes; however, few models capture their combined effects. We developed a deterministic ordinary differential equation model stratified by age and deprivation decile coupled with time-dependent testing proportion to examine how mixing patterns shape inequalities in disease burden, using COVID-19 in England as a case study. The framework allows three mixing scenarios–diagonal, preferred, and proportionate, and we simulated the epidemic with movement restrictions to reflect lockdown measures. We assessed the effectiveness of these restrictions in reducing transmission and explored their implications for different social groups. Results show that under diagonal mixing, disease outcomes are significantly higher than under the other mixing scenarios, with the most deprived deciles experiencing disproportionately greater burdens. Lockdown measures substantially reduced overall disease outcomes across all deciles, but relative inequalities persisted, indicating that generalised restrictions alone were insufficient to eliminate these inequalities. Our age–deprivation structured model provides a foundation for designing targeted interventions, such as deprivation-focused testing, vaccination campaigns, or localised movement restrictions, to mitigate unequal health outcomes during epidemics.

## 1 Introduction

In late 2019, the world experienced a new virus, the Severe Acute Respiratory Syndrome Coronavirus 2 (SARS-CoV-2), of pandemic magnitude that redefined how people worked and responded to public health emergencies. During the global spread of SARS-CoV-2, non-pharmaceutical interventions (NPIs) were widely implemented measures to reduce virus transmission. Measures such as stay-at-home orders, social distancing, hand washing, school closure, closure of social and religious centres, and mask wearing were some of the first NPIs implemented, after which other pharmaceutical interventions such as vaccination and management drugs were adopted to slow down COVID-19 transmission globally [1]. Spinelli et al. [2] demonstrated that the differences in the incidence and severity of COVID-19 between countries can be attributed to the population’s reaction to the implemented NPIs.

Although the virus had a global impact, differential outcomes were reported across sociodemographic groups [3–5] highlighting that the COVID-19 pandemic affected different parts of the population disproportionately. A notable instance is the disproportionately high rate of COVID-19 infection, hospitalisation, and mortality among racial and ethnic minorities [4]. Ethnic minorities, despite making up only 14% of the population in the UK, account for 34% of those critically ill [6]. This differential outcome could be attributed to disparities in healthcare access and socioeconomic opportunities between majority and minority ethnic groups. Therefore, it is crucial to examine the differential impacts of the pandemic on various sociodemographic groups to enable policymakers to identify and address these disparities effectively. We sought to examine how social mixing patterns influenced the varied impacts of the COVID-19 pandemic across different sociodemographic groups, using England, United Kingdom (UK), as a case study. We do not seek to replicate the events of the pandemic in England; rather, we present a modelling framework that could be useful for decision-making in the future.

In this study, sociodemographic groups were defined using the 2019 Index of Multiple Deprivation (IMD) [7], which serves as a recognised metric for assessing relative deprivation in England. Research has suggested that social and health inequalities are associated with the level of deprivation [8], and Bach-Mortensen and Degli [9] showed that deprivation is a major risk factor for the mortality rate of COVID-19. Such differences in COVID-19 outcomes based on deprivation levels may stem from economic, social, and health inequalities.

For instance, the population of people in the most deprived groups may face challenges working remotely, rely more on public transport, and may experience increased contact in workplace settings compared to those in the least deprived groups, thus increasing their risk of contracting a disease such as COVID-19. To reduce these disparities, it is necessary to identify factors such as age, occupation, and income that increase risk within each deprived group. As a result, infectious disease models have become increasingly important for policymakers in developing solutions to problems related to disease outbreaks and epidemiological issues, especially during endemic or epidemic health crises [10]. Mathematical models have provided epidemiological insights and have been used to estimate epidemic size, incidence, infection peak, and the impact of intervention policies.

Some mathematical models have been used to highlight the significance of age in disease spread, both in terms of case incidence and disease severity during the pandemic [11–13]. Disparities in the severity and mortality of the virus based on age [11]. In addition, susceptibility, transmissibility, hospitalisation rates, and death rates have been shown to be age-dependent [12,14,15]. Davies et al. [12] revealed that the susceptibility of individuals under 20 years of age to coronavirus disease is approximately half that of adults aged over 20 years. Furthermore, it was found that clinical symptoms (requiring hospital care) occurred in 21% of infections in 10-19 year olds, compared to 69% of infections in people aged over 70 years. The study also suggested that interventions targeted at children may have a limited effect on reducing SARS-CoV-2 transmission, particularly if the subclinical transmissibility of the infection is low [12]. The impact of the COVID-19 pandemic varied across different communities and individuals. Densely populated areas and jobs requiring frequent contact (healthcare and service industries) increased infection risk, particularly in lower socioeconomic status (SES) communities. Manna et al. [16] introduced a model that creates synthetic generalised contact matrices that incorporate age and SES, such as income, employment, and gender, for epidemic modelling. A study by Manna et al. [17] in Hungary demonstrated that employment status and education significantly affected contact rates and vaccination acceptance, highlighting the role of social determinants in epidemic-related behaviour. Hale et al. [18] used the 2019 English Index of Multiple Deprivation (IMD) to segregate the population based on deprivation levels, showing that variations in social mixing, testing/reporting motivation, and precautionary measures (e.g. mask-wearing and vaccination) contributed to differences in daily contact rates between the most and least deprived groups.

Also, previous modelling studies have shown that both age and socioeconomic status (SES) are critical determinants of infectious disease burden. Goodfellow et al. integrated age and deprivation deciles to model COVID-19 in England, finding that uniform policies disproportionately benefited affluent groups, thereby widening inequalities [19]. Ma et al. examined race and ethnicity as proxies for SES in New York and demonstrated that preferential within-group mixing amplified disparities in infection [20]. Similarly, Gozzi et al. used mobility data in Chile to show that wealthier districts reduced contacts more during lockdown, leaving poorer areas with higher attack and death rates [21], while earlier influenza models of Kumar et al. and Hyder and Leung highlighted that both population composition and social segregation were necessary to reproduce observed inequalities [22,23].

Building on these insights, our study contributes uniquely by combining age and deprivation decile into an explicitly stratified framework, systematically varying mixing assumptions from diagonal to proportionate, and incorporating a time-dependent testing proportion that reflects early pandemic conditions using England as a case study. Together, these features enable us to capture the complex interactions between age and socioeconomic status, and to reveal how disparities in transmission and outcomes emerge from their intersection.

In this study, we present a deterministic mathematical modelling framework that couples age and deprivation mixing contact structure together with a time-dependent testing rate to analyse disease dynamics across different age and deprivation deciles. This study aimed to understand the effect of social mixing within and between deprivation deciles on disease dynamics, using the COVID-19 pandemic in England as a case study. The findings provide insight into the mechanisms that result in disproportionate disease outcomes between different sociodemographic groups during the outbreak of infectious diseases, such as the COVID-19 pandemic.

## 2 Materials and methods

### 2.1 Model description

In this study, we present a deterministic ordinary differential equation model to capture disease transmission and control. The mathematical model framework integrated sociodemographic and age group interactions with time-varying testing rates. Each epidemiological stage was divided using an extended compartmental SEIR-type mathematical model. Sociodemographic groups are defined as deprivation deciles based on the Index of Multiple Deprivation (IMD) in England, which is a measure extrapolated from seven domains of deprivation: income, employment, education, health, crime, barriers to housing and services, and living environment [7]—and used to define social groups at the lower-layer Super Output Area (LSOA) level or neighbourhood. Four or five groups of output areas (OAs), each comprising 40–250 households, make up the LSOAs [24]. A typical LSOA includes between 400 and 1,200 households, with a resident population of between 1,000 and 3,000 [24].

It is important to note that the aim of this study was not to replicate the exact course of the COVID-19 pandemic in England, but to build a framework capturing disparities across age and deprivation groups. To ensure plausibility, parameters were taken from published studies and publicly available data (see Table 1). Contact rates were derived from the POLYMOD [25] study and scaled for lockdown, while testing dynamics were calibrated using ONS data (January–September 2020) as shown in the S1 File. Where no direct estimates existed, plausible assumptions were applied.

**Table 1:** Model parameter values and references.

Parameter	Description	Value & Source
*c*_*i*,*j*_	contact matrix by age group *i* and *j*	[25]
*ι*	Modification factor for transmission by confirmed/reported cases	0.6 (assumed)
ρ	Proportion of cases that are asymptomatic	0.6 (assumed)
*θ*	Proportion of population that are symptomatic	0.4 [26]
*q*	Relative infection rate per contact	2.5 (assumed)
*γ*	recovery rate	$1/14\;days^{-1}$ [26]
$\delta_{i}$	The rate of symptomatic or positive tested individuals become hospitalised, varies by age.	[13]
*d*_*i*_	The rate at which hospitalised individuals become fatally ill. This varies by age	[13]
ϵ	Rate of recovered individuals become susceptible (waning immunity)	$2/100\;days^{-1}$ [27]
*a*_*i*_	Infectivity by age	[13]
*h*_*i*_	susceptibility by age	[13]
σ	Rate at which exposed individuals become infectious (latent period)	1/3 days^−1^ (assumed)

The indices of multiple deprivation (IMD) have (32,844) distinct categories in England, ranked from the most deprived group (1) to the least deprived group (32,844). The deprived groups were sub-divided into ten equal segments to form deprivation deciles. To parameterise the model, we used demographic data from the Office for National Statistics (ONS) dataset ‘Monthly populations by Index of Multiple Deprivation (IMD) decile, England [28]’ This dataset provides population denominators by both IMD decile and age group, ensuring that our stratification reflects the actual demographic distribution in England. Each decile was further stratified into 21 age bands (0–4 through 90+ years).

In this mathematical model framework, each deprivation decile has 21-age classes segregated by 5-year age band: 0-4, 5-9,..., 90+. The model has eight compartments. susceptible ($S_{i}^{n}$), exposed ($E_{i}^{n}$), asymptomatic ($A_{i}^{n}$), undetected and detected symptomatic ($J_{i}^{n}$), $I_{i}^{n}$ respectively, hospitalised ($H_{i}^{n}$), recovered ($R_{i}^{n}$), and dead ($D_{i}^{n}$) individuals. The notation (*i*) and (*n*) represents individuals in age group *i* and deprivation decile *n*.

In this study, the model was set up such that susceptible individuals contacted infectious individuals at rate ($\beta_{i}^{n}S_{i}^{n}/N_{i}^{n}$), a function of social mixing, age mixing, susceptibility and infectivity by age, and relative transmission rate. Upon contact with infectious individuals, they transition into the exposed compartment. After exposure, individuals can progress to the asymptomatic, undetected, and detected symptomatic compartments after a latent period of (*σ* ). Detection of symptomatic individuals is dependent on the testing rate (π(t)), such that in an event where less testing is done, more individuals would become undetected, and some could become asymptomatic at a rate ($\sigma (1\;-\;\pi (t))\rho E_{i}^{n}$) or symptomatic at a rate ($\sigma (1\;-\;\pi (t))\theta E_{i}^{n}$). Individuals in the population have the same infection period of (*γ*), and symptomatic individuals can become hospitalised at a rate ($\delta_{i}$). However, we assumed that only hospitalised individuals could die from their infection at a rate (*d*_*i*_). In this study, we assumed that the testing capacity grows over time and is very low at the beginning of an outbreak, aligning with the study of [18]. The parameter π(t) is the testing capacity defined by the Generalised Richards Model (GRM) proposed by [29]. The cumulative number of tests per day is defined in Eq (1) as

$$\frac{\text{d}\pi }{\text{dt}}=r[\pi (0){]}^{p}\left(1-{\left(\frac{\pi (t)}{K}\right)}^{\alpha }\right)$$ (1)

where *r* is the growth rate, *K* is the carrying capacity, *p* is the growth profile (that is, if p=α=1 we would have the classical logistic growth model), *α* is the deviation away from the S-shape of the logistic curve, and π(0) is the initial number of tests. Here, we assume that the test rate is uniform and the same for the entire population. We estimated the parameters (details in S1 File) r,p,K, &α using the non-linear least square method, by fitting to the UK COVID-19 cumulative number of tests data from 3 January to 3 September 2020 of the publicly available data [30]. This study considered the period when the wild-type SARS-CoV-2 was dominant in the UK and the period of the first lockdown.

**Fig 1:**
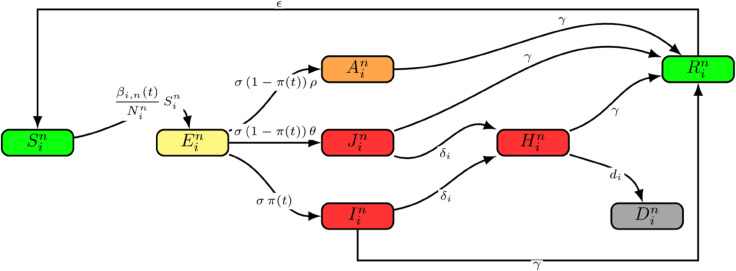
The epidemiological model of mixing within and between age groups and deprivation deciles.

The model equations for Fig 1 are a system of differential equations for individuals in age group *i* and social group *n*, presented as:

$$\frac{dS_{i}^{n}}{dt}=-(\beta {)}_{i,n}(t)\frac{S_{i}^{n}}{N_{i}^{n}}+\epsilon R_{i}^{n}$$ (2a)

$$\frac{dE_{i}^{n}}{dt}=(\beta {)}_{i,n}(t)\frac{S_{i}^{n}}{N_{i}^{n}}-\sigma \left[\pi (t)+(1-\pi (t))\rho +(1-\pi (t))\theta \right]E_{i}^{n}$$ (2b)

$$\frac{dA_{i}^{n}}{dt}=\sigma (1-\pi (t))\rho E_{i}^{n}-\gamma A_{i}^{n}$$ (2c)

$$\frac{dJ_{i}^{n}}{dt}=\sigma (1-\pi (t))\theta E_{i}^{n}-\delta_{i}J_{i}^{n}-\gamma J_{i}^{n}$$ (2d)

$$\frac{dI_{i}^{n}}{dt}=\sigma \pi (t)E_{i}^{n}-\delta_{i}I_{i}^{n}-\gamma I_{i}^{n}$$ (2e)

$$\frac{dH_{i}^{n}}{dt}=\delta_{i}J_{i}^{n}+\delta_{i}I_{i}^{n}-\gamma H_{i}^{n}-d_{i}H_{i}^{n}$$ (2f)

$$\frac{dR_{i}^{n}}{dt}=\left(I_{i}^{n}+A_{i}^{n}+J_{i}^{n}+H_{i}^{n}\right)\gamma -\epsilon R_{i}^{n}$$ (2g)

$$\frac{dD_{i}^{n}}{dt}=d_{i}H_{i}^{n}$$ (2h)

where θ=1−ρ, and π(t)+(1−π(t))ρ+(1−π(t))θ=1. The force of infection is defined as the effective contact between and within deprivation deciles (while maintaining constant age mixing) that produces an infection. Previous studies have shown the differential impact of age on the dynamics of coronavirus disease 2019 (COVID-19) [13,31,32]. Mathematically, we define the force of infection as

$$(\beta {)}_{i,n}(t)=\sum_{k=1}^{10}\sum_{j=1}^{21}\lambda_{i,n,j,k}\left(J_{j,k}+\iota I_{j,k}+A_{j,k}\right)$$ (3)

and,

$$\lambda_{i,n,j,k}=\hat{q}\left(w_{n,k}\cdot {\tilde{c}}_{i,j}\right),$$ (4)

where,

$${\tilde{c}}_{i,j}=\left(c_{i,j}\cdot (a_{i}\cdot h_{j})\right).$$ (5)

The *c*_*i*,*j*_ in Eq (5) is the average number of contacts per day from POLYMOD data [25], *a*_*i*_ the age-related differential infectivity, *h*_*i*_ the age-related differential susceptibility, and $\hat{q}$ is the relative transmission rate. This study used scaled POLYMOD data for England segmented into 21 age groups (5-year age bands) as used by [13]. Age contacts are divided into an average number of contacts per day at home, school, work, and other places. The total number of contacts is defined as the sum of contacts at home, school, work, and other places:

$$c_{i,j}^{T}=\Omega_{1}c_{i,j}^{H}+\Omega_{2}c_{i,j}^{S}+\Omega_{3}c_{i,j}^{W}+\Omega_{4}c_{i,j}^{O}$$ (6)

where $\Omega_{1-4}$ in Eq (6) are the scaling factors for age mixing during the lockdown period. In this study, movement restrictions were modelled to approximate the first national lockdown in England. Contact matrices were adjusted such that household contacts were preserved, school and other community contacts were reduced to near zero, and workplace contacts were substantially reduced but not eliminated, reflecting the continued activity of essential workers (e.g., healthcare and supermarket staff). This simplified approach was designed to capture the general effect of lockdown on mixing patterns, rather than to reproduce the full sequence of policy changes during the pandemic. More details on lockdown implementation is in the S1 File.

The mixing pattern by deprivation decile (*w*_*n*,*k*_ ) in Eq (4) is defined by the equation proposed by [33]. Each element *w*_*n*,*k*_ is the average number of contacts made within and between the deprivation deciles, given as

$$w_{n,k}=\left(\frac{\epsilon \nu_{n}\nu_{k}}{D}+\frac{(1-\epsilon )\nu_{n}\delta_{n,k}}{N_{n}}\right)$$ (7)

where $\left(\delta_{n,k}:k=k\;is\;1,\;\&\;n\neq k\;is\;0\right)$, ϵ∈[0,1], and $\nu_{n}$ is the average number of contacts by each sociodemographic group, *N*_*n*_ is the total population by decile, after aggregating by age groups. $D=\sum_{n=1}\sum_{i=1}N_{n}^{i}$ is the total number of contacts per unit time made by all individuals in the population. Each *w*_*n*,*k*_ is generated by normalising Eq (7) so that the sum of the matrix *w*_*n*,*k*_ is equal to 1.

To our knowledge, there are no known estimated values for social mixing patterns by deciles of deprivation. Studies, such as [18,20,33] that studied mixing between different demographic groups had to make some assumptions about the mixing pattern of the population under study. We made a simplistic assumption that the average number of contacts in each sociodemographic group decreased linearly from 10 to 1, with 10 representing the daily average number of interactions for the most deprived group and 1 for the least deprived group. We then adjusted the value of *ε* to examine the various mixing patterns.

The mixing assumptions defined using Eq (7), where the parameter ϵ∈[0,1] controls the proportion of within-decile versus between-decile mixing. We considered three scenarios: (i) **Diagonal mixing** (ϵ=0), where individuals interact only within their own deprivation decile and groups evolve independently; (ii) **Preferred mixing** (0<ϵ<1), where most interactions occur within a decile but a fraction occur between deciles (we considered ϵ=0.3 and ϵ=0.7); and (iii) **Proportionate mixing** (ϵ=1), where contacts are random across the whole population regardless of deprivation decile. These scenarios represent, respectively, highly localised contact structures, partially stratified contact patterns, and fully global contact structures. Their implications are that stronger within-decile mixing magnifies health inequalities by concentrating transmission in deprived groups, whereas proportionate mixing diffuses risk more evenly but still reflects demographic disparities.

Our aim was to investigate the impact of different mixing patterns on disease outcomes in different sociodemographic groups. To achieve this, we considered the diagonal (people mix only within their groups and nowhere else), preferred (people are more likely to interact with those in their own group), and proportionate (people mix randomly with the entire population) mixing scenarios using the defined mixing assumptions.

## 3 Results

In this study, parameters were selected from the existing literature (see Table 1) and simulated for 200 days to mimic the period in England when the wild-type SARS-CoV-2 virus was still dominant and the period when the first lockdown and easing was implemented. This choice reflects the study’s focus on understanding disparities during the early phase of the pandemic rather than the full course of events. While longer simulations would affect the absolute magnitude of outcomes, the relative disparities between deprivation deciles and age groups observed here are qualitatively robust. Future applications of this framework could extend the time horizon to incorporate vaccination, subsequent waves, and the emergence of new variants. At *t* = 0, we assumed that a detected infectious individual entered the system I(0)=1 in the most deprived group and age group 55–59. This assumption was maintained for all mixing scenarios (0<ϵ≤1), when ϵ=0, we further assumed that an infectious individual I(0)=1 was detected across all deciles of deprivation in the same age groups. The focus was to understand the infection dynamics between different mixing scenarios by observing the total number of infections (detected and undetected), number of detected cases, hospitalised individuals, and deaths across deprivation deciles. The findings illustrate the impact of integrating different mixing scenarios on disease dynamics, both within and across deprivation deciles. In this analysis, the cumulative number of infections at any specific time (*t*) was determined by summing the individuals in the detected symptomatic and undetected symptomatic and asymptomatic compartments. Detected individuals are those who had tested positive for the SARS-CoV-2 virus after taking a test and were considered confirmed cases. The proportion of the population tested was estimated using the generalised Richards model presented in Eq (1) fitted to the cumulative test data during the COVID-19 pandemic.

### 3.1 Diagonal mixing scenario

In this study, a diagonal mixing scenario is defined when ϵ=0 in Eq (7). This scenario highlights local mixing, where mixing interaction is dominant exclusively with individuals within your decile, without any interaction with individuals from different deciles. This results in a “within-group homogeneous" mixing pattern, where each group operates independently of the others, indicating that the individuals within each decile mix evenly and possess similar traits. Consequently, Eq (3) can be expressed as

$$(\beta {)}_{n,i}(t)=\sum_{j=1}^{21}\lambda_{i,n,j,n}\left({𝐉}_{j,n}+\iota {𝐈}_{j,n}+\tau {𝐀}_{j,n}\right)$$ (8)

and,

$$\lambda_{i,n,j,n}=\hat{q}\left(w_{n,n}\cdot {\tilde{c}}_{i,j}\right).$$ (9)

However, the force of infection captures mixing exclusively in deprivation deciles. Eq (8) demonstrates that the growth of infection occurs independently within each group because there is no interaction between the groups; only within-group dynamics are observed. Since there was no interaction between deciles, we initialised our model by introducing an infection into each decile and followed our initial assumption that the most deprived groups tended to mix more frequently than people in the least deprived groups. This assumption about social mixing behaviour is reasonable considering that people in the most deprived groups are likely to have more interactions owing to factors such as greater reliance on public transportation, larger household size, and types of employment [18].

**Fig 2:**
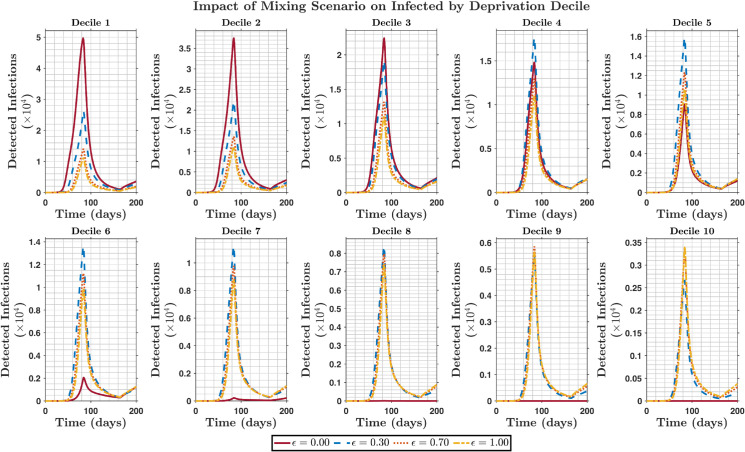
Comparison of detected infectious individual by deprivation decile between different mixing scenario (0≤ϵ≤1). The red line is the time series of detected infectious individuals for the diagonal mixing assumption (ϵ=0). The blue dashed and red dotted lines are the preferred (ϵ=0.3 and ϵ=0.7) and proportionate (ϵ=1) mixing assumptions, respectively, represented by the yellow dashed line by deprivation decile. This figure illustrates how different social mixing patterns affect the infection burden across deprivation levels, with disproportionately higher infection outcomes in most deprived deciles, compared to the least deprived groups.

This study does not seek to replicate the COVID-19 pandemic but aims to create a framework that is beneficial for future pandemic planning. Rigorous mathematical and sensitivity analyses of the model are presented in the S1 File. In the diagonal mixing scenario, the most deprived groups exhibited significantly higher infection outcomes than the least deprived groups, which demonstrated limited or no infection outcomes, as shown in Fig 2. The groups exhibiting a notably high number of infections were those with a sufficient average number of contacts that could produce secondary infections, while groups with significantly lower or no infections were those in which interactions were low enough to not allow secondary infections. Because this scenario considered interaction solely within the deprivation decile, the value of *R*_0_ must be greater than 1 in each decile for the disease to spread (or generate secondary infections).

This mixing scenario works better in communities where people mostly interact within their social, economic, or sociodemographic groups, such as rural areas, segregated societies, or areas with cultural or economic disparities. The diagonal mixing assumption is more suitable for highly stratified societies where cross-dimensional transmission is rare.

### 3.2 Preferred mixing assumption

This mixing scenario is suitable for environments where mixing is neither fully global nor fully local, as it enables us to capture the dual interaction of inter and intra groups. The preferred mixing scenario demonstrates both local and global mixing interactions, such that there is a higher likelihood of contacting individuals in your social group (local interaction) than randomly meeting people outside your social group (global interaction). In this study, we compared two variations of *ε*: (i) When ϵ=0.3: a stronger mixing or interaction within groups and significantly minimal mixing between groups can be observed. This is because from Eq (7), 70% of interactions will occur within deciles, and the remaining 30% will occur between deciles. (ii) ϵ=0.7 implies that only 30% of interactions occur within deciles compared to the 70% interactions between deciles.

This type of interaction can be applied to different types of social interactions in the human population. For example, someone in an executive position who is most likely to be in the least deprived group is more likely to interact frequently with people in the least deprived group and less likely to interact with people from the most deprived group. Hence, this type of setup captures the strong mixing within the main diagonal and linearly decreasing interactions outside the main diagonal. This implies that the farther an individual is from a group, the less likely they are to interact with individuals from that group.

### Proportionate mixing assumption

At ϵ=1 in Eq (7), the scenario for proportionate mixing, where the mixing within and between sociodemographic groups are equally likely. That is, there is pure global mixing, meaning people can interact with anyone without considering social domain or status. This approach is possible in a highly connected, cosmopolitan environment where barriers to interaction are minimal; for example, in universities, where people from diverse backgrounds interact. This form of mixing can facilitate interactions that resemble “spill-over" between groups without a specific focus on connecting with more individuals from one’s own demographic group. In addition, when ϵ=1, Eq (7) only considers the quadratic part; therefore, there is no forcing on the main diagonal. This implies that the values along the main diagonal are squared by the average number of contacts in each group and multiplied by the average number of contacts with other groups off-diagonal. Consequently, contact decreases as it moves away from the most deprived group towards the least deprived group, but that does not necessarily mean that the mixing along the diagonal is greater than the mixing off the diagonal.

It is worthy of note that, under the lockdown assumptions implemented, overall infection, hospitalisation, and mortality outcomes decreased substantially across all deprivation deciles. However, relative inequalities persisted, with the most deprived groups continuing to experience a disproportionately higher burden of disease. This suggests that while generalised movement restrictions are effective in reducing transmission, they do not by themselves eliminate the structural disparities in outcomes between sociodemographic groups in our model.

## Discussion

In this study, we presented a modelling framework that combined age and deprivation decile mixing coupled with time-varying test rates to understand COVID-19 outcomes, such as infection, hospitalisation, and deaths, using the population of England as a case study. Sensitivity analysis of key parameters and mathematical analysis to validate if model assumptions make epidemiological sense are presented in the S1 File. Our findings revealed that diagonal mixing resulted in a higher number of disease outcomes than other types of mixing assumptions. All mixing assumptions presented agree that the most deprived group has a disproportionately higher rate of infection outcome (incidence, hospitalisation, and mortality) during the outbreak. This suggests that both socioeconomic status and social interaction patterns are critical factors influencing disease transmission and its severity.

Previous research [16–18,21,34–36] has emphasised the potential influence of sociodemographic groups such as age, education, and income on the outcomes of infectious diseases. In recent times, only a few of these studies [16–18] have coupled age and other sociodemographic groups into a mathematical model to understand the dynamics of infectious diseases, and of [18] used the deprivation decile. We presented a deterministic modelling approach to capture social interaction within and between deprivation deciles, coupled with time-varying testing rates. Our findings align with the mathematical model results of [18], who presented a shift known as “deprivation switching" in their work. This shift involved the least deprived group exhibiting higher infection outcomes than the most deprived group during spring 2021, before switching back to the most deprived group exhibiting a higher infection outcome similar to the earlier stage of the pandemic. This phenomenon could be explained as either a change in reporting behaviour across deprivation groups or increased variation in social mixing across deprivation deciles [18].

**Fig 3:**
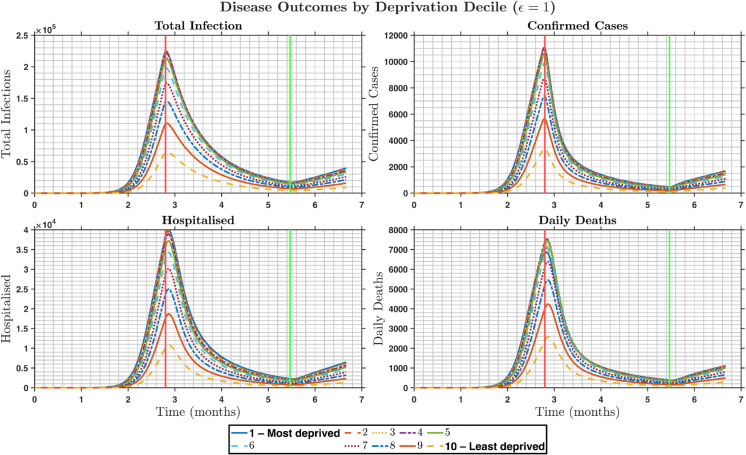
Time series by deprivation decile of total infections (undetected + detected), detected cases, hospitalisations, and deaths under the “preferred” mixing assumptions ϵ=0.3. **Top left panel** The total infections by deprivation decile, **Top right panel** the number of detected cases, **Bottom right panel** the number of hospitalised individuals, **Bottom left panel** the number of daily deaths. This panel shows that—with 70% of contacts occurring within the same decile—more deprived groups experience both earlier and larger peaks in all outcomes.

In our study, the disease outcomes in the diagonal mixing scenario were higher than those in the other mixing scenarios, as shown in Fig 2. In the preferred mixing scenario, where similar groups tend to engage with each other frequently, the high-activity group, which represents the most disadvantaged group in our case, will predominantly interact within its own group rather than with other groups. This leads to notably elevated rates of disease outcomes within these groups, as illustrated in Fig 3 when ϵ=0.3. Thus, promoting the focus of control efforts on the most deprived groups will be crucial. This is because if public health authorities could achieve herd immunity within the most deprived groups, the value of *R*_0_ in that group would fall below 1, and the capacity to spread secondary infections would decrease. As a result, the probability of infection spillover to other deprivation deciles is ultimately reduced, benefitting the overall population. The proportionate mixing scenario would require a different type of control effort because the likelihood of interaction between social groups is the same, and the control efforts would need to be targeted to the entire population. Hence, interventions are more expensive and sometimes difficult to achieve.

Figs 3 and 5 present four key epidemic outcomes over time for each deprivation decile but under different mixing scenarios. In Fig 3 (ϵ=0.3), there are strong within-decile interactions causing concentrated transmission in the most deprived groups, resulting in sharp, early peaks in the first three deciles and minimal outbreaks in the last three deciles. In Fig 5 where ϵ=1.0, pure global mixing forced the distribution of infection more evenly, causing the peak of the most deprived groups to drop by approximately 30−−40%, while the least deprived groups exhibited slightly higher peaks compared with the preferred-mixing scenario. This highlights the fact that preferred mixing intensifies inequality in the infection burden by concentrated mixing within groups, whereas proportionate mixing reduces disparities between deciles by trading intensity in deprived groups for a broader spread into less deprived ones.

**Fig 4:**
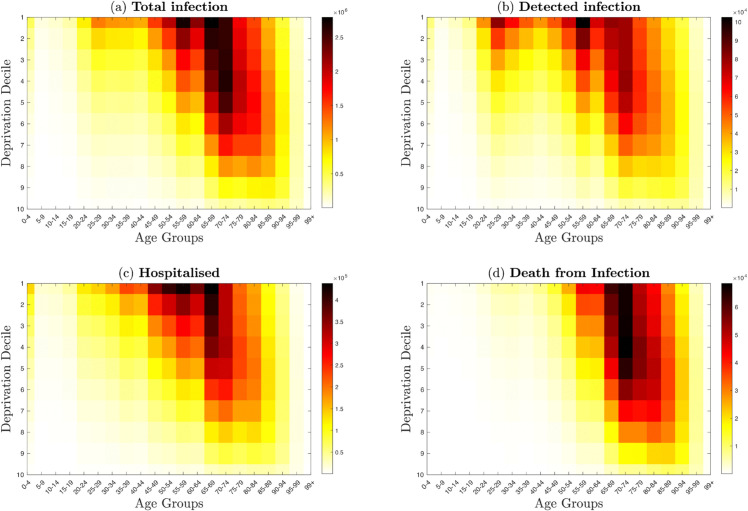
Cumulative burden across age groups and deprivation deciles. **Top left panel:** sum of the total number of infection aggregated by age and deprivation decile. **Top right panel:** sum of detected number of detected cases aggregated by age and deprivation decile. **Bottom right panel:** The sum of hospitalised individuals by age and deprivation decile. **Bottom left panel** The sum of daily death by age and deprivation decile. This figure highlights the unequal burden of COVID-19 outcomes across age and deprivation groups at ϵ=0.3. Most deprived groups and older ages bear a disproportionately large share of severe outcomes, highlighting the compound effect of socioeconomic deprivation and age.

**Fig 5:**
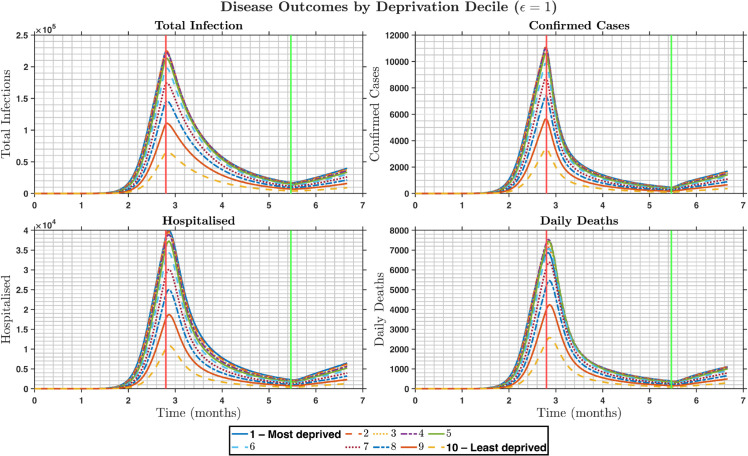
Time series of total infections (undetected and detected), detected cases, hospitalisations, and deaths individuals by deprivation decile, during the lockdown and easing period at ϵ=1. **Top left panel** The total number of infections by deprivation decile, **Top right panel** the number of detected cases, **Bottom right panel** the number of hospitalised individuals, **Bottom left panel** the number of daily deaths.

**Fig 6:**
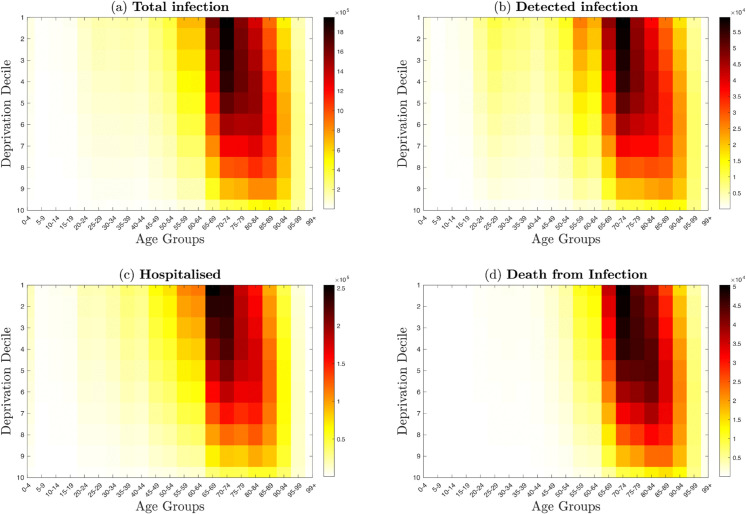
Cumulative outcomes across age bands and deprivation deciles at ϵ=1. **Top left panel:** sum of the total infection aggregated by age and deprivation decile. **Top right panel:** sum of the daily number of detected cases aggregated by age and deprivation decile. **Bottom right panel:** The sum of hospitalised individuals by age and deprivation decile. **Bottom left panel** The sum of daily death by age and deprivation decile. The overall burden is more evenly distributed by decile than in the preferred-mixing case, though older age groups remain at highest risk across all deprivation levels.

Fig 4 aggregates outcomes by age and decile at ϵ=0.3, revealing that the joint effects of high deprivation and old age drive the largest cumulative burdens, particularly in older adults within the most deprived groups. In contrast, Fig 6 shows that while age remains the dominant risk factor for severe outcomes, the disease outcome by decile gradient is much shallower: the least deprived (deciles 8–10) also accumulate substantial infections and hospitalisations over time. In other words, preferred mixing leads to “hotspots” of age and deprivation, whereas proportionate mixing produces a more uniform age-stratified curve across all deciles.

The diagonal mixing approach can provide an inaccurate, underestimated, or overestimated view of the actual burden of the disease in each group, since although individuals usually interact within their social groups, there will still be some interactions with individuals from other groups that the diagonal mixing assumption did not consider. Although both diagonal and proportionate mixing models would require control efforts targeted at the entire population, less effort and resources would be required to control disease outbreaks in proportionate mixing. This is because the entire population interacts, and herd immunity would be swiftly reached. In comparison, diagonal mixing requires herd immunity to be achieved within each group to effectively control disease spread.

In contrast to prior research, our model enables simultaneous inference of disease dynamics across social and age groups. By utilising our model framework, it would be possible to observe the infection outcome by age and deprivation decile simultaneously, as shown in Figs 3 and 5. This would be important in knowing which age group to target for intervention in each deprivation decile, hence helping to maximise resources. Fitting data to this study would have made the results more interesting; however, we provided a mathematical and sensitivity analysis to validate some of our model assumptions.

Sensitivity analysis (analytic and numeric) confirmed that contact patterns are the dominant driver of *R*_0_ and disparities in infection outcomes. A uniform scaling of the contact matrix has an elasticity of 1, that is, a 1% increase in overall mixing raises *R*_0_ by 1% (see S1 Fig), with individual mixing entries contributing heterogeneously across the matrix. We also found significant sensitivity to the recovery rate *γ* and hospitalisation rate $\delta_{i}$, since shorter infectious periods and faster hospital removal reduce *R*_0_. The asymptomatic fraction *ρ* was another important driver, emphasising the role of hidden transmission in infection outcome. By contrast, sensitivities to the testing rate *π* and modification factor *ι* were smaller (see S1 Fig, S2 Fig); given limited testing capacity early in the pandemic. These results indicate that controlling contacts, shortening infectious duration, and removing hospitalised cases are crucial for mitigating disparities in infection outcomes.

Our findings have practical implications for public health policy and decision making. Although broad restrictions such as movement restrictions/lockdowns reduce overall transmission, the model shows that relative disparities persist, with deprived groups bearing a disproportionate burden. This suggests that targeted interventions are needed to complement general measures. For example, tailored workplace protections for essential workers could reduce the high infection outcomes experienced in these groups. Enhanced support for testing and isolation in deprived communities is also crucial. Additionally, prioritisation of vaccination or other preventive resources for high-risk groups could further mitigate the unequal burden of infections. By systematically varying mixing structures and incorporating time-dependent testing, the framework also provides a tool for policymakers to anticipate how social segregation and detection capacity shape inequalities, and to design strategies that address both overall control and equity objectives.

Although we lack access to data that capture the daily number of infections by age and deprivation decile in England, having access to such data would substantially strengthen this work, as model fitting approach could help us estimate the average number of contacts within deprivation deciles, thus providing better insight into disease dynamics by decile. The main objective of this research is to showcase how mixing patterns impact disease progression across deprivation deciles. Consequently, we postulate that the assumption of diagonal mixing is similar to homogeneous mixing within different social groups; however, it offers a straightforward method to collectively model the dynamics within these groups.

This study has several limitations. First, it was not intended to replicate the exact trajectory of COVID-19 in England but to provide a general framework for exploring disparities across age and deprivation groups. Second, empirical estimates of social mixing by deprivation decile were not available, and we therefore applied a linear assumption for contact rates based on the study of [18], and modulated by different mixing scenarios. Third, the implementation of lockdown was simplified by preserving household contacts, reducing school and community contacts to near zero, and retaining minimal workplace contacts to reflect essential workers. This approach was appropriate as the study focused on the period of the first lockdown and its easing in England. Future studies could extend this framework to incorporate the more complex and evolving restrictions that occurred over longer periods of the pandemic. Fourth, testing was modelled using a Generalised Richards growth model with a uniform testing proportions across the population, which does not capture local variations in capacity or uptake. If granular data becomes available to capture local variation, our framework would be useful in understanding the population heterogeneity in testing. Also, we understand that fitting this data cumulatively can create a smoothing effect in some cases, but it is a standard approach that is regularly used in epidemiology when fitting noisy data [37,38].

Finally, we assumed that only hospitalised individuals could die. We acknowledge that this does not capture the full spectrum of outcomes during the pandemic, but it is a reasonable simplification for representing the earlier phase of COVID-19 when severe cases were primarily managed in hospitals. In addition, several parameters (for example, relative transmission rate, modification factor for detected cases) were assumed in the absence of direct estimates. While these assumptions may influence quantitative outputs, they do not change the key qualitative conclusion that stronger within-group mixing and deprivation structures amplify inequalities in disease outcomes.

This study makes a unique contribution by explicitly modelling the intersection of age and deprivation. In our framework, a susceptible individual in age group *i* and deprivation decile *n* can acquire infection from individuals in age group *j* and decile *k*, allowing disparities to emerge directly from the combined effects of demographic age structure and deprivation gradients. Second, we systematically vary social mixing across deprivation deciles—from diagonal (within-decile) through preferred to proportionate—to reveal how the degree of social segregation (low *ε*) versus homogeneous mixing (high *ε*) mechanistically shapes inequalities. Finally, we incorporate a time-dependent testing rate π(t), which captures the early ramp-up of testing capacity and its dynamic effect in reducing undetected transmission.

Although the model uses COVID-19 as case study, the age–deprivation structured framework is general and could be extended to other infectious diseases or health outcomes. By adjusting natural history parameters and transmission routes, the model can represent pathogens such as influenza, RSV, or tuberculosis. Also, the same structure could be used to study disparities in vaccination uptake, chronic disease risks, or access to healthcare, where outcomes are jointly shaped by demographic age structure and socioeconomic segregation. Explicitly representing the intersection of age and deprivation, and varying mixing structures, offers a flexible tool for exploring how health inequalities emerge and persist across different public health contexts.

In conclusion, this study investigated the influence of social mixing patterns on disease outcomes in heterogeneous populations. The results indicate a significantly higher rate of infection among the most deprived compared to the least deprived groups. Our model can facilitate the implementation of tailored interventions targeting the most affected sociodemographic groups in the future. This is achievable through the use of deprivation deciles as a proxy for sociodemographic groups, which indirectly enables spatial intervention due to the location-based nature of deciles, as opposed to using income or ethnicity which can be more broadly representative of the entire population. We developed a deterministic ordinary differential equation with a multi-layered structured model to mimic COVID-19 disease outcomes, focusing on the total number of infections, detected cases, hospitalised individuals, and deaths in the population. We have provided a significant contribution by creating a mathematical model that considers age and sociodemographic mixing coupled with time-dependent testing rate, along with time-sensitive non-pharmaceutical interventions (such as lockdowns) to monitor the progression of a disease after control measures are put in place. The incorporated dynamic testing rate, similar to logistic growth, can be useful in understanding early infection growth during a new outbreak. This methodology could be applied in future studies to better understand the collective impact of age and socioeconomic status on disease spread. Additionally, our work will motivate future research that would want to understand the combined effect of age and other sociodemographic mixing infection dynamics, and how intervention can be better tailored to avoid wastage in the presence of different mixing assumptions.

## Supporting information

S1 File Supplementary methods and analyses. (PDF)

S1 Fig Bar chart of normalised sensitivity indices. Each bar represents the relative impact of changes in the model parameters, with positive values indicating parameters that increase *R*0 and negative values indicating those that decrease it.
(TIF)

S2 FigTime-varying basic reproduction number *R*_0_(*t*) under different testing rate trajectories π(t).(TIF)

## References

[1] Brodeur A, Grigoryeva I, Kattan L. Stay-at-home orders, social distancing, and trust. J Popul Econ. 2021;34(4):1321–54. doi: 10.1007/s00148-021-00848-z 34177123 PMC8214058

[2] Spinelli MA, Glidden DV, Gennatas ED, Bielecki M, Beyrer C, Rutherford G, et al. Importance of non-pharmaceutical interventions in lowering the viral inoculum to reduce susceptibility to infection by SARS-CoV-2 and potentially disease severity. Lancet Infect Dis. 2021;21(9):e296–301. doi: 10.1016/S1473-3099(20)30982-8 33631099 PMC7906703

[3] Bajwah S, Edmonds P, Yorganci E, Chester R, Russell K, Lovell N, et al. The association between ethnicity, socioeconomic deprivation and receipt of hospital-based palliative care for people with Covid-19: a dual centre service evaluation. Palliat Med. 2021;35(8):1514–8. doi: 10.1177/02692163211022959 34098811

[4] Lo C-H, Nguyen LH, Drew DA, Warner ET, Joshi AD, Graham MS, et al. Race, ethnicity, community-level socioeconomic factors, and risk of COVID-19 in the United States and the United Kingdom. EClinicalMedicine. 2021;38:101029. doi: 10.1016/j.eclinm.2021.101029 34308322 PMC8285255

[5] Valdes AM, Moon JC, Vijay A, Chaturvedi N, Norrish A, Ikram A, et al. Longitudinal assessment of symptoms and risk of SARS-CoV-2 infection in healthcare workers across 5 hospitals to understand ethnic differences in infection risk. EClinicalMedicine. 2021;34:100835. doi: 10.1016/j.eclinm.2021.100835 33880438 PMC8049191

[6] Soltan MA, Varney J, Sutton B, Melville CR, Lugg ST, Parekh D, et al. COVID-19 admission risk tools should include multiethnic age structures, multimorbidity and deprivation metrics for air pollution, household overcrowding, housing quality and adult skills. BMJ Open Respir Res. 2021;8(1):e000951. doi: 10.1136/bmjresp-2021-000951 34373239 PMC8354812

[7] English indices of deprivation 2019 . Ministry of Housing CaLG. 2019. https://www.gov.uk/government/statistics/english-indices-of-deprivation-2019

[8] Kulkarni K, Shah R, Mangwani J, Dias J. The impact of deprivation on patients awaiting planned care. Bone Jt Open. 2022;3(10):777–85. doi: 10.1302/2633-1462.310.BJO-2022-0037.R1 36210732 PMC9626867

[9] Bach-Mortensen AM, Degli Esposti M. Is area deprivation associated with greater impacts of COVID-19 in care homes across England? A preliminary analysis of COVID-19 outbreaks and deaths. J Epidemiol Community Health. 2021;75(7):624–7. doi: 10.1136/jech-2020-215039 33558430

[10] Dembek ZF, Chekol T, Wu A. Best practice assessment of disease modelling for infectious disease outbreaks. Epidemiol Infect. 2018;146(10):1207–15. doi: 10.1017/S095026881800119X 29734964 PMC9134297

[11] Zou L, Ruan F, Huang M, Liang L, Huang H, Hong Z, et al. SARS-CoV-2 viral load in upper respiratory specimens of infected patients. N Engl J Med. 2020;382(12):1177–9. doi: 10.1056/NEJMc2001737 32074444 PMC7121626

[12] Davies NG, Klepac P, Liu Y, Prem K, Jit M, CMMID COVID-19 working group, et al. Age-dependent effects in the transmission and control of COVID-19 epidemics. Nat Med. 2020;26(8):1205–11. doi: 10.1038/s41591-020-0962-9 32546824

[13] Keeling MJ, Guyver-Fletcher G, Dyson L, Tildesley MJ, Hill EM, Medley GF. Precautionary breaks: planned, limited duration circuit breaks to control the prevalence of SARS-CoV2 and the burden of COVID-19 disease. Epidemics. 2021;37:100526. doi: 10.1016/j.epidem.2021.100526 34875583 PMC8636324

[14] Zhang J, Litvinova M, Liang Y, Wang Y, Wang W, Zhao S, et al. Changes in contact patterns shape the dynamics of the COVID-19 outbreak in China. Science. 2020;368(6498):1481–6. doi: 10.1126/science.abb8001 32350060 PMC7199529

[15] Eastin C, Eastin T. Epidemiological characteristics of 2143 pediatric patients with 2019 coronavirus disease in China. The Journal of Emergency Medicine. 2020;58(4):712–3. doi: 10.1016/j.jemermed.2020.04.006

[16] Manna A, Dall’Amico L, Tizzoni M, Karsai M, Perra N. Generalized contact matrices allow integrating socioeconomic variables into epidemic models. Sci Adv. 2024;10(41):eadk4606. doi: 10.1126/sciadv.adk4606 39392883 PMC11468902

[17] Manna A, Koltai J, Karsai M. Importance of social inequalities to contact patterns, vaccine uptake, and epidemic dynamics. Nat Commun. 2024;15(1):4137. doi: 10.1038/s41467-024-48332-y 38755162 PMC11099065

[18] Hale AC, Read JM, Jewell CP. Modelling the impact of social mixing and behaviour on infectious disease transmission: application to SARS-CoV-2. 2022. https://arxiv.org/abs/2211.02371

[19] Goodfellow L, van Leeuwen E, Eggo RM. COVID-19 inequalities in England: a mathematical modelling study of transmission risk and clinical vulnerability by socioeconomic status. BMC Med. 2024;22(1):162. doi: 10.1186/s12916-024-03387-y 38616257 PMC11380352

[20] Ma KC, Menkir TF, Kissler S, Grad YH, Lipsitch M. Modeling the impact of racial and ethnic disparities on COVID-19 epidemic dynamics. Elife. 2021;10:e66601. doi: 10.7554/eLife.66601 34003112 PMC8221808

[21] Gozzi N, Tizzoni M, Chinazzi M, Ferres L, Vespignani A, Perra N. Estimating the effect of social inequalities on the mitigation of COVID-19 across communities in Santiago de Chile. Nat Commun. 2021;12(1):2429. doi: 10.1038/s41467-021-22601-6 33893279 PMC8065143

[22] Kumar S, Piper K, Galloway DD, Hadler JL, Grefenstette JJ. Is population structure sufficient to generate area-level inequalities in influenza rates? An examination using agent-based models. BMC Public Health. 2015;15:947. doi: 10.1186/s12889-015-2284-2 26400564 PMC4579639

[23] Hyder A, Leung B. Social deprivation and burden of influenza: testing hypotheses and gaining insights from a simulation model for the spread of influenza. Epidemics. 2015;11:71–9. doi: 10.1016/j.epidem.2015.03.004 25979284

[24] ONS. Census 2021 geographies. https://www.ons.gov.uk/methodology/geography/ukgeographies/censusgeographies/census2021geographies#: :text=Lower

[25] Mossong J, Hens N, Jit M, Beutels P, Auranen K, Mikolajczyk R, et al. Social contacts and mixing patterns relevant to the spread of infectious diseases. PLoS Med. 2008;5(3):e74. doi: 10.1371/journal.pmed.0050074 18366252 PMC2270306

[26] Muller K, Muller PA. Mathematical modelling of the spread of COVID-19 on a university campus. Infect Dis Model. 2021;6:1025–45. doi: 10.1016/j.idm.2021.08.004 34414342 PMC8364150

[27] Anggriani N, Beay LK. Modeling of COVID-19 spread with self-isolation at home and hospitalized classes. Results Phys. 2022;36:105378. doi: 10.1016/j.rinp.2022.105378 35280116 PMC8896885

[28] Office for National Statistics. Monthly populations by Index of Multiple Deprivation (IMD) decile, England: January 2019 to August 2022. 2022. https://www.ons.gov.uk/peoplepopulationandcommunity/populationandmigration/populationprojections/adhocs/15363monthlypopulationsbyindexofmultipled/eprivationimddecileenglandjanuary2019toaugust2022

[29] Wu K, Darcet D, Wang Q, Sornette D. Generalized logistic growth modeling of the COVID-19 outbreak: comparing the dynamics in the 29 provinces in China and in the rest of the world. Nonlinear Dyn. 2020;101(3):1561–81. doi: 10.1007/s11071-020-05862-6 32836822 PMC7437112

[30] UK Government. Coronavirus (COVID-19) in the UK. 2023. https://coronavirus.data.gov.uk/details/testing?areaType=nation&areaName=England

[31] Franco N, Coletti P, Willem L, Angeli L, Lajot A, Abrams S, et al. Inferring age-specific differences in susceptibility to and infectiousness upon SARS-CoV-2 infection based on Belgian social contact data. PLoS Comput Biol. 2022;18(3):e1009965. doi: 10.1371/journal.pcbi.1009965 PMC9000131 35353810

[32] Ram V, Schaposnik LP. A modified age-structured SIR model for COVID-19 type viruses. Sci Rep. 2021;11(1):15194. doi: 10.1038/s41598-021-94609-3 34312473 PMC8313685

[33] Hethcote HW. Modeling heterogeneous mixing in infectious disease dynamics. In: Models for infectious human diseases: their structure and relation to data. vol. 215; 1996. p. 238.

[34] Abda A, Del Giorgio F, Gauvin L, Autmizguine J, Kakkar F, Drouin O. Association between area-level material deprivation and incidence of hospitalization among children with SARS-CoV-2 in Montreal. Paediatr Child Health. 2022;27(Suppl 1):S27–32. doi: 10.1093/pch/pxab106 35620560 PMC9126283

[35] Mariné Barjoan E, Chaarana A, Festraëts J, Géloen C, Prouvost-Keller B, Legueult K, et al. Impact of social and demographic factors on the spread of the SARS-CoV-2 epidemic in the town of Nice. BMC Public Health. 2023;23(1):1098. doi: 10.1186/s12889-023-15917-z 37280635 PMC10243248

[36] Fortunato F, Lillini R, Martinelli D, Iannelli G, Ascatigno L, Casanova G, et al. Association of socio-economic deprivation with COVID-19 incidence and fatality during the first wave of the pandemic in Italy: lessons learned from a local register-based study. Int J Health Geogr. 2023;22(1):10. doi: 10.1186/s12942-023-00332-9 37143110 PMC10157567

[37] Betti MI, Heffernan JM. A simple model for fitting mild, severe, and known cases during an epidemic with an application to the current SARS-CoV-2 pandemic. Infect Dis Model. 2021;6:313–23. doi: 10.1016/j.idm.2021.01.002 33521406 PMC7833529

[38] Whitaker SA, Golightly A, Gillespie CS, Kypraios T. Sequential bayesian inference for stochastic epidemic models of cumulative incidence. Bayesian Anal. 2025;1(1):1–30. doi: 10.1214/25-ba1547

