# Supplementary material for: Differential impacts of the COVID-19 pandemic on sociodemographic groups: A mathematical model framework
Source: PLoS One. 2026 Jan 27;21(1):e0330273. doi: 10.1371/journal.pone.0330273 (PMC12844531; doi:10.1371/journal.pone.0330273)
Supplement: S1 File — (PDF) [file pone.0330273.s001.pdf]

Supplementary material to: Differential Impacts of the COVID-19 Pandemic on Sociodemographic Groups: A Mathematical Model Framework

* gbeminiyi.oyedele@warwick.ac.uk

Appendix A Mathematical Model Formulation

A.1 Model Equations

The system of differential equations for the population in age group i and social group n is as follows:

$$\frac{dS_i^n}{dt} = -(\boldsymbol{\beta})_{i,n}(t)\frac{S_i^n}{N_i^n} + \epsilon R_i^n \tag{1a}$$

$$\frac{dE_i^n}{dt} = (\boldsymbol{\beta})_{i,n}(t)\frac{S_i^n}{N_i^n} - \sigma\left[\pi(t) + (1-\pi(t))\rho + (1-\pi(t))\theta\right] E_i^n \tag{1b}$$

$$\frac{dA_i^n}{dt} = \sigma(1-\pi(t))\rho E_i^n - \gamma A_i^n \tag{1c}$$

$$\frac{dJ_i^n}{dt} = \sigma(1-\pi(t))\theta E_i^n - \delta_i J_i^n - \gamma J_i^n \tag{1d}$$

$$\frac{dI_i^n}{dt} = \sigma\pi(t)E_i^n - \delta_i I_i^n - \gamma I_i^n \tag{1e}$$

$$\frac{dH_i^n}{dt} = \delta_i J_i^n + \delta_i I_i^n - \gamma H_i^n - d_i H_i^n \tag{1f}$$

$$\frac{dR_i^n}{dt} = \left(I_i^n + A_i^n + J_i^n + H_i^n\right)\gamma - \epsilon R_i^n \tag{1g}$$

$$\frac{dD_i^n}{dt} = d_i H_i^n \tag{1h}$$

where $\theta = 1-\rho$, and $\pi(t) + (1-\pi(t))\rho + (1-\pi(t))\theta = 1$. Here, $(\boldsymbol{\beta})_{i,n}(t)$ is defined as

$$(\boldsymbol{\beta})_{i,n}(t) = \sum_{k=1}^{10}\sum_{j=1}^{21} \boldsymbol{\lambda}_{i,n,j,k}\left(J_{j,k} + \iota I_{j,k} + A_{j,k}\right) \tag{2}$$

where $\boldsymbol{\lambda}_{i,n,j,k}$ is the contact rate between individuals in age group i and social group n and those in age group j and social group k as shown in Figure 1.

$$N_i^n = S_i^n + E_i^n + A_i^n + J_i^n + I_i^n + H_i^n + D_i^n + R_i^n. \tag{3}$$

Additionally, $\boldsymbol{\lambda}_{i,n,j,k}$ is the contact rate between individuals in age group i and social group n and those in age group j and social group k. Furthermore, $\boldsymbol{\lambda}_{i,n,j,k}$ are elements of the block matrix $\boldsymbol{\lambda}$, which is a square matrix of size $n \times n$, where $n = 1, 2, \ldots, 10$ and each element is a matrix of size $i, j = 1, 2, \ldots, 21$. In this model, we assume that only symptomatic and asymptomatic individuals $\mathbf{J}_{n,j}, \mathbf{I}_{n,j},$ and $, \mathbf{A}_{n,j}$ contribute directly to infection transmission, and that there is no transmission of infection in hospitals. In addition, we assume that asymptomatic

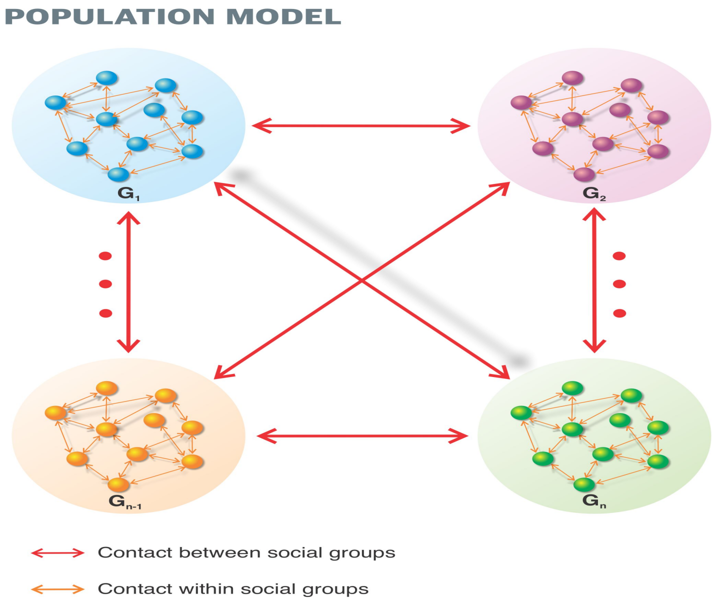

Fig 1. Population interactions by age and deprivation deciles. $G_1 \ldots G_n$ are sociodemograhic groups which equate to deprivation deciles.

individuals can transmit the disease without being aware of it, so we do not impose a modification factor for the transmission of asymptomatic individuals.

In equation (2), we define

$$\boldsymbol{\lambda}_{i,n,j,k} = \hat{q}\left(w_{n,k} \cdot c_{i,j}\right), \tag{4}$$

where $\mathbf{C}$ is the age contact mixing matrix from the study of Mossong *et al.* [1], $\hat{q}$ is the relative transmission rate, and the social interaction by deprivation decile is derived using the method of Hethcote [2], being defined as

$$w_{n,m} = \left(\frac{\epsilon \nu_n \nu_m}{D} + \frac{(1-\epsilon)\nu_n \delta_{n,m}}{N_n}\right) \tag{5}$$

where $(\delta_{n,m} : m = m \; is \; 1, \; \& \; n \neq m \; is \; 0)$, $\epsilon \in [0,1]$, and ν_n are the average number of contacts for each sociodemographic group. Here, n and m are the deprivation deciles, N_n is the total population of each decile in England, $D = \Sigma_{n=1} \nu_n N_n$ is the total number of contacts per unit time for everyone in the population.

A.1.1 Key model assumptions for mathematical analysis

- The preferred mixing method ($\epsilon = 0.3$ in equation 5) was adopted for interactions between deprivation deciles for the purpose of the mathematical analysis.
- It was assumed that similar age mixing [1] occurs consistently across these deciles.
- The age–deprivation contact matrix is held constant over the simulated period in the mathematical analysis, not in the main text. This omits temporal changes in behaviour (lockdowns, school schedules, holidays) or adaptive responses to rising cases.
- Births, natural deaths, ageing between groups, and migration are ignored over the simulated periods, so population sizes in each compartment change only via disease transitions.

- rate of recovery (γ), hospitalisation (δ_i), and death (d_i) are uniform across deciles. We assumed that deprivation affects only the contact rates.

Appendix B Testing Fraction $\pi(t)$

In the model, exposed individuals can progress to the asymptomatic A_i^n, undetected or detected symptomatic compartment J_i^n or I_i^n respectively, the detected symptomatic individuals are assumed to have been tested for disease at rate $\pi(t)$ after taking a test (PCR or lateral flow) and move to compartment I_i^n. In this study, we make the assumption that the testing capacity grows over time, and is very low at the start of the outbreak, this aligns will with the result of [3]. Parameter $\pi(t)$, which is the testing capacity is defined by the Generalised Richards Model (GRM) proposed by [4], is defined in equation (6).

The cumulative number of tests by time is given as:

$$\frac{d\pi}{dt} = r[\pi(t)]^p \left(1 - \left(\frac{\pi(t)}{K}\right)^\alpha\right) \tag{6}$$

where r is the growth rate of testing (that is the rate at which testing becomes available), K is the carrying capacity (the maximum testing level), p is the growth profile (that is, it controls how growth depends on the current testing).

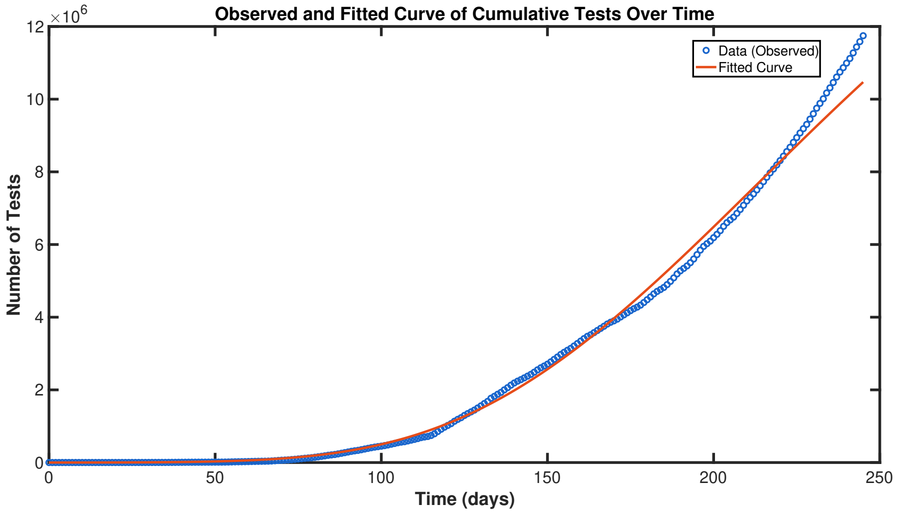

Fig 2. The blue line shows the cumulative number of tests reported for SARS-COV-2 between January, 03 and September, 03 2020 from publicly available UK data, and the red line shows the fitted curve using the Levenberg-Marquardt algorithm for least squares parameter estimation.

If $p = \alpha = 1$ we would have the classical logistic growth model, but if $p < 1$ implies faster initial growth.The parameter α controls the symmetry of the S-shape; $\alpha < 1$ speeds up the saturation, and $\alpha > 1$ delays the saturation. $\pi(t)$ is the number of tests at time t. We estimated the parameters $r, p, K, \ \& \alpha$ using the Non-linear least square method, by fitting to the UK COVID-19 cumulative number of tests data from January, 03 - September, 03 2020 of the publicly available data [5]. This study considered the period when the wild-type of SARS-CoV2 was dominant in the UK, and the period of the first lockdown.

The time-dependent function $\pi(t) \in [0,1]$ represents the fraction of exposed individuals who are detected at the point of progressing to infectiousness. All individuals leave the exposed compartment at rate σ (1/time); a fraction $\pi(t)$ enter the detected infectious class, while the remaining $1-\pi(t)$ enter the undetected class. Thus, σ supplies the temporal units, and $\pi(t)$ acts as a unitless partition of this flow.

B.1 Lockdown Implementation

Restriction	Description	value
Total Lockdown	Closing non-essential places like pubs and supermarkets boosts home mixing and lowers workplace mixing. Home mixing adjusts for higher household transmission during lockdown by a factor Ω_1, while Ω_3 indicates minimal workplace mixing.	$c^T_{i,j} = c^H_{i,j}\Omega_1 + c^W_{i,j}\Omega_3$
Easing	As more non-essential places and workplaces open, workplace interaction increases, scaling up the parameter Ω_3, and some minimal interaction in other places Ω_2.	$c^T_{i,j} = c^H_{i,j}\Omega_1 + c^W_{i,j}\Omega_3 + c^O_{i,j}\Omega_4$
Relaxed Restriction	Everything that happened during easing of lockdown with the addition of minimised mixing at other places by the scaling parameter Ω_4. The removal of restrictions will mean that all locations are accessible again, however, the rate of mixing has not yet returned to what it was prior to the lockdown.	$c^T_{i,j} = c^H_{i,j}\Omega_1 + c^W_{i,j}\Omega_3 + c^S_{i,j}\Omega_2 + c^O_{i,j}\Omega_4$

Table 1. Lockdown implementation by age groups.

Our aim was not to replicate the COVID-19 transmission event as it happened in England, but to provide a framework that would be beneficial to future pandemic studies. This allows us to make the simplified assumption that lockdown reduced socio-contact mixing by 50% within the decile of deprivation. This was to capture the impact of lockdown on employment-related contact across the deprivation decile; it was assumed that essential workers who remained physically present at work may contribute to ongoing transmission. Deprivation is known to be correlated with age [3], allowing us to focus more on the lockdown strategy within the age-mixing matrix. The implementation of the age restriction is presented in Table 1.

Appendix C Analytical solutions

We analysed our model analytically to verify whether the model system (1) behaves consistently with epidemiological intuition.

C.1 Basic Qualitative Properties of Model 1

In this subsection, we prove that the solution to system (1) is mathematically and biologically relevant if and only if it is positive and bounded.

Theorem 1 (Positivity)**.** *Let* $\Omega = \Big\{(S_i^n, E_i^n, A_i^n, J_i^n, I_i^n, H_i^n, R_i^n, D_i^n) \in \mathbb{R}_+^8 : N_i^n \leq \mathbf{N_0} : S_i^n(0) \geq 0, E_i^n(0) \geq 0, A_i^n(0) \geq 0, J_i^n(0) \geq 0, I_i^n(0) \geq 0, H_i^n(0) \geq 0, R_i^n(0) \geq 0, D_i^n(0) \geq 0\Big\}$*. Then, the solution* $(S_i^n(t), E_i^n(t), A_i^n(t), J_i^n(t), I_i^n(t), H_i^n(t), R_i^n(t), D_i^n(t))$ *of system (1) remains positive for all* $t > 0$.

Proof. The initial conditions $S_i^n(0) \geq 0, E_i^n(0) \geq 0, A_i^n(0) \geq 0, J_i^n(0) \geq 0, Q_i^n(0) \geq 0, I_i^n(0) \geq 0, H_i^n(0) \geq 0, R_i^n(0) \geq 0, D_i^n(0) \geq 0$ will be used to prove the positivity of the system (1). From equation (1a), we let

$$H = \frac{\beta_{n,i}}{N_i^n}, \tag{7}$$

Equation (1a) can now be written as

$$\frac{dS_i^n}{dt} + HS_i^n = \epsilon R_i^n. \tag{8}$$

The integrating factor of equation (8) is given by

$$\mathbf{IF} = e^{\int Hds}.$$

We multiply equation (8) by **IF**:

$$e^{\int_0^t Hds}\frac{dS_i^n}{dt} + e^{\int_0^t Hds} HS_i^n = \epsilon R_i^n \cdot e^{\int_0^t Hds}. \tag{9}$$

In equation (9), we observe that the LHS is the derivative of **IF** multiplied by S_i^n. Hence, we can write equation (9) as:

$$\frac{d}{dt}[e^{\int_0^t Hds} S_i^n] = \epsilon R_i^n \cdot e^{\int_0^t Hds}. \tag{10}$$

Taking the derivative of both sides, we obtain

$$\int_0^t \frac{d}{dt}\left(S_i^n(u) e^{\int_0^u Hds} S\right) du = \int_0^t \epsilon R(u) \cdot e^{\int_0^u Hds} du.$$

$$\implies S(t) e^{\int_0^t Hds} - S_i^n(0) = \int_0^t \epsilon R_i^n(u) \cdot e^{\int_0^u Hds} du.$$

Further rearranging yields

$$S_i^n(t) = S_i^n(0) \cdot e^{-\int_0^t Hds} + \int_0^t \epsilon R_i^n(u) \cdot e^{\int_u^t Hds} du. \tag{11}$$

From the initial conditions, we know that $S_i^n \geq 0$ means that $S_i^n(0) \cdot e^{-\int_0^t Hds} > 0$ and all parameters are positive. That is, $\epsilon > 0$, and hence, $\int_0^t \epsilon R_i^n(u) \cdot e^{\int_u^t Hds} du > 0$. Therefore, we can conclude that S_i^n is positive for all $t > 0$. The same is true for $E_i^n(0) \geq 0$, $A_i^n(0) \geq 0$, $J_i^n(0) \geq 0$, $Q_i^n(0) \geq 0$, $I_i^n(0) \geq 0$, $H_i^n(0) \geq 0$, $R_i^n(0) \geq 0$ and $D_i^n(0) \geq 0$.

□

Theorem 2 (boundedness)**.** *All feasible solutions of the model system (1) are uniformly bounded in a closed set* $\Omega = \left\{(S_i^n, E_i^n, A_i^n, J_i^n, I_i^n, H_i^n, R_i^n, D_i^n) \in \mathbb{R}_+^8 : N_i^n(0) \leq \mathbf{N_0}\right\}$.

Proof. At $t = 0$, $S_i^n(0) \geq 0$, $E_i^n(0) \geq 0$, $A_i^n(0) \geq 0$, $J_i^n(0) \geq 0$, $Q_i^n(0) \geq 0$, $I_i^n(0) \geq 0$, $H_i^n(0) \geq 0$, $R_i^n(0) \geq 0$, and $D_i^n(0) \geq 0$. Considering the boundedness of the system (1) under the initial conditions such that $S_i^n(t) + E_i^n(t) + A_i^n(t) + J_i^n(t) + I_i^n(t) + H_i^n(t) + R_i^n(t) + D_i^n(t) = N_i^n(t)$, we have

$$\frac{dN_i^n}{dt} = \frac{dS_i^n}{dt} + \frac{dE_i^n}{dt} + \frac{dA_i^n}{dt} + \frac{dJ_i^n}{dt} + \frac{dI_i^n}{dt} + \frac{dH_i^n}{dt} + \frac{dR_i^n}{dt} + \frac{dD_i^n}{dt}.$$

By taking the sum of the system of ordinary differential equations, we obtain

$$\frac{dN_i^n}{dt} = 0.$$

This implies that N_i^n remains constant over time. By integrating both sides with respect to t and applying the initial condition $N_i^n(0) = \mathbf{N_0}$, we obtain $N_i^n(0) \leq \mathbf{N_0}$ for all t. Hence, the feasible solution of the model system remains in the region $\Omega = \left\{(S_i^n, E_i^n, A_i^n, J_i^n, I_i^n, H_i^n, R_i^n, D_i^n) \in \mathbb{R}_+^8 : N_i^n(0) \leq \mathbf{N_0}\right\}$.

□

C.2 Disease-Free Equilibrium and Basic Reproduction Number

The basic reproduction number is the average number of infected individuals in a completely susceptible population. Considering the system of equations (1a–1h) and the total population in equation (3), the infected compartments are E_i^n (exposed), A_i^n (asymptomatic), J_i^n (undetected symptomatic), and I_i^n (detected symptomatic). The compartments A_i^n, J_i^n, and I_i^n contribute directly to the force of infection, whereas E_i^n is the point at which a susceptible individual comes into contact with an infected individual. In the disease-free equilibrium (DFE), $E_i^n = A_i^n = J_i^n = I_i^n = H_i^n = D_i^n = R_i^n = 0$ and $S_i^n = N_i^n$.

We solve for R_0 using the next-generation matrix approach and then determine the dominant eigenvalue of $(\mathcal{T}\mathcal{V}^{-1})$. We divide the system into disease transmission $(\mathcal{T})$ and disease transition $(\mathcal{V}^{-1})$, as follows:

$$\mathcal{T} = \begin{bmatrix} \sum_{k=1}^{n}\sum_{j=1}^{i} \boldsymbol{\lambda}_{i,n,j,k}\left(\mathbf{J}_{n,j} + \iota\mathbf{I}_{n,j} + \mathbf{A}_{n,j}\right)\frac{S_i^n}{N_i^n} \\ 0 \\ 0 \\ 0 \\ 0 \end{bmatrix},$$

$$\mathcal{V} = \begin{bmatrix} \sigma E_i^n \\ -\sigma(1-\pi)\rho E_i^n + \gamma A_i^n \\ -\sigma(1-\pi)\theta E_i^n + (\delta_i + \gamma)J_i^n \\ -\sigma\pi E_i^n + (\delta_i + \gamma)I_i^n \end{bmatrix}.$$

The Jacobian of the two systems at the DFE and for $\theta = 1 - \rho$ is calculated as

$$\mathcal{T} = \begin{bmatrix} 0 & \sum_{k=1}^{n}\sum_{j=1}^{i}\boldsymbol{\lambda}_{i,n,j,k} & \sum_{k=1}^{n}\sum_{j=1}^{i}\boldsymbol{\lambda}_{i,n,j,k} & \sum_{k=1}^{n}\sum_{j=1}^{i}\iota\boldsymbol{\lambda}_{i,n,j,k} \\ 0 & 0 & 0 & 0 \\ 0 & 0 & 0 & 0 \\ 0 & 0 & 0 & 0 \\ 0 & 0 & 0 & 0 \end{bmatrix}$$

$$\mathcal{V} = \begin{bmatrix} \sigma & 0 & 0 & 0 \\ -\sigma(1-\pi)\rho & \gamma & 0 & 0 \\ -\sigma(1-\pi)\theta & 0 & \delta_i + \gamma & 0 \\ -\sigma\pi & 0 & 0 & \delta_i + \gamma \end{bmatrix}.$$

The inverse of $\mathcal{V}$ is given by

$$\mathcal{V}^{-1} = \begin{bmatrix} \frac{1}{\sigma} & 0 & 0 & 0 \\ \frac{(1-\pi)\rho}{\gamma} & \frac{1}{\gamma} & 0 & 0 \\ \frac{(1-\pi)\theta}{\delta_i+\gamma} & 0 & \frac{1}{\delta_i+\gamma} & 0 \\ \frac{\pi}{\delta_i+\gamma} & 0 & 0 & \frac{1}{\delta_i+\gamma} \end{bmatrix}.$$

Hence, we can compute $\mathcal{TV}^{-1}$ for one group as

$$\mathcal{TV}^{-1} = \left[0, \boldsymbol{\lambda}_{n,j}\left(\frac{(1-\pi)\rho}{\gamma} + \frac{(1-\pi)\theta}{\delta_i+\gamma} + \frac{\iota\pi}{\delta_i+\gamma}\right), 0, 0\right]. \tag{12}$$

For multiple groups, we construct a full matrix

$$R_0 = \sum_{k=1}^{n}\sum_{j=1}^{i} \boldsymbol{\lambda}_{i,n,j,k}\left(\frac{(1-\pi)\rho}{\gamma} + \frac{(1-\pi)(1-\rho)}{\delta_j+\gamma} + \frac{\iota\pi}{\delta_j+\gamma}\right), \tag{13}$$

From equation (13), R_0 is largely dependent on the mixing matrix (which is the combination of age and deprivation mixing).

C.3 Stability of the Disease-Free Equilibrium

Theorem 3 (local stability)**.** *The DFE of system (1) is locally asymptotically stable (LAS) if $R_0 < 1$ and unstable if $R_0 > 1$.*

Proof. To demonstrate the local stability of our model, we linearise the system by finding the Jacobian at the DFE. The Jacobian matrix at the DFE, where $S_i^n = N_i^n$ and all other compartments are zero, is an 8×8 matrix

$$\mathbf{J} = \begin{pmatrix} 0 & 0 & -\beta^* & -\beta^* & -\iota\beta^* & 0 & \epsilon & 0 \\ 0 & -\sigma & \beta^* & \beta^* & \iota\beta^* & 0 & 0 & 0 \\ 0 & \sigma(1-\pi)\rho & -\gamma & 0 & 0 & 0 & 0 & 0 \\ 0 & \sigma(1-\pi)\theta & 0 & -(\delta_i+\gamma) & 0 & 0 & 0 & 0 \\ 0 & \sigma\pi & 0 & 0 & -(\delta_i+\gamma) & 0 & 0 & 0 \\ 0 & 0 & 0 & \delta_i & \delta_i & -(\gamma+d_i) & 0 & 0 \\ 0 & 0 & \gamma & \gamma & \gamma & \gamma & -\epsilon & 0 \\ 0 & 0 & 0 & 0 & 0 & d_i & 0 & 0 \end{pmatrix}. \tag{14}$$

For simplicity, let $\beta^* = \sum_{k=1}^{10}\sum_{j=1}^{21} \boldsymbol{\lambda}_{i,n,j,k}$. In this Jacobian matrix, the only nonzero entries in the first and last rows are ϵ and d_i, representing the rates at which individuals lose their immunity to become susceptible and die from a severe infection, respectively. These rates are negligible at DFE, as there are no infections, recoveries, or disease-related deaths. This implies that the impact of susceptibility loss due to infection exposure and infection-related death on the dynamics of other compartments is minimal. Hence, we focus on disease dynamics by removing the S_i^n and D_i^n compartments and reducing the system to six compartments: E_i^n, A_i^n, J_i^n, I_i^n, H_i^n, and

R_i^n. The differential equations for these compartments at DFE are

$$\mathbf{J} = \begin{pmatrix} -\sigma & \beta^* & \beta^* & \iota\beta^* & 0 & 0 \\ \sigma(1-\pi)\rho & -\gamma & 0 & 0 & 0 & 0 \\ \sigma(1-\pi)\theta & 0 & -\delta_i-\gamma & 0 & 0 & 0 \\ \sigma\pi & 0 & 0 & -(\delta_i+\gamma) & 0 & 0 \\ 0 & 0 & \delta_i & \delta_i & -\gamma-d_i & 0 \\ 0 & \gamma & \gamma & \gamma & \gamma & -\epsilon \end{pmatrix}. \tag{15}$$

We obtain the characteristic equation by setting $|J - \lambda I|$ to zero:

$$|J-\lambda I| = \begin{vmatrix} -\sigma-\lambda & \beta^* & \beta^* & \iota\beta^* & 0 & 0 \\ \sigma(1-\pi)\rho & -\gamma-\lambda & 0 & 0 & 0 & 0 \\ \sigma(1-\pi)\theta & 0 & -\delta_i-\gamma-\lambda & 0 & 0 & 0 \\ \sigma\pi & 0 & 0 & -\delta_i-\gamma-\lambda & 0 & 0 \\ 0 & 0 & \delta_i & \delta_i & -\gamma-d_i-\lambda & 0 \\ 0 & \gamma & \gamma & \gamma & \gamma & -\epsilon-\lambda \end{vmatrix} = 0. \tag{16}$$

Hence, the coefficient of the characteristic polynomial is given by

$$\mathbf{F}(\lambda) = \lambda^7 + a_6\lambda^6 + a_5\lambda^5 + a_4\lambda^4 + a_3\lambda^3 + a_2\lambda^2 + a\lambda + a_0 = 0 \tag{17}$$

where

$$a_6 = 1$$

$$a_5 = \sigma + 4\gamma + 2\delta_i + d_i + \epsilon$$

$$\begin{aligned} a_4 =& 7\gamma^2 + 8\gamma\delta_i + 4\sigma\gamma + 3\sigma\delta_i + \delta_i^2 + 3d_i\delta_i + 3\epsilon\delta_i + \sigma d_i + 3\gamma d_i + \sigma\epsilon + 4\gamma\epsilon + 2d_i\epsilon \\ &- \lambda_{n,i}\sigma(1-\pi)\rho - \lambda_{n,i}\sigma(1-\pi)(1-\rho) - \iota\lambda_{n,i}\sigma\pi \end{aligned}$$

$$\begin{aligned} a_3 =& 4\gamma^3 + (6\epsilon + 6\sigma + 3d_i + \delta_i)\gamma^2 + 5\gamma^2\delta_i + (4\epsilon + 6\sigma + d_i)\gamma\delta_i + 2\gamma\delta_i^2 \\ &+ (2\epsilon + 3\sigma + 2d_i + 2\delta_i)\sigma\gamma + (2\epsilon + 2\sigma + d_i)d_i\delta_i + (\epsilon + \sigma + d_i)\delta_i^2 + (\epsilon + \sigma)d_i\sigma + \epsilon\sigma\delta_i \\ &- \sigma\lambda_{n,i}\left[(1-\pi)\rho(\epsilon + 3\gamma + d_i + 2\delta_i) + (1-\pi)(1-\rho)(\epsilon + 3\gamma + d_i + \delta_i) + \iota\pi(\epsilon + 3\gamma + d_i + \delta_i)\right] \end{aligned}$$

$$\begin{aligned} a_2 =& 6\gamma^4 + 11\gamma^3\delta_i + (6\sigma + 3d_i + 2\epsilon)\gamma^3 + (8\sigma + 4d_i + 5\epsilon + 2\delta_i)\gamma^2\delta_i + (4\sigma + 2d_i + \epsilon + \delta_i)\gamma\delta_i^2 + (d_i + \epsilon)\delta_i^3 \\ &+ (6\epsilon\sigma + 4\sigma^2 + 4\sigma d_i + 2\sigma\delta_i + 2\epsilon d_i + \epsilon\delta_i)\gamma^2 + (4\epsilon\sigma + 2\sigma^2 + 3\sigma d_i + 3\sigma\delta_i + \epsilon d_i)\gamma\delta_i \\ &+ (\sigma^2 + \sigma d_i + \sigma\delta_i)d_i\delta_i + (\sigma d_i + \sigma\delta_i)\delta_i^2 + (\epsilon\sigma + \sigma^2)d_i^2 + \epsilon\sigma^2\gamma + \epsilon\sigma\delta_i^2 \\ &- \sigma\lambda_{n,i}\big[(1-\pi)\rho(5\gamma^2 + 5\gamma\delta_i + 2d_i\gamma + 2d_i\delta_i + 2\epsilon\gamma + \epsilon d_i + \epsilon\delta_i) \\ &+ (1-\pi)(1-\rho)(3\gamma^2 + 2\gamma\delta_i + d_i\gamma + d_i\delta_i + 2\epsilon\gamma + \epsilon d_i + \epsilon\delta_i) \\ &+ \iota\pi(3\gamma^2 + 2\gamma\delta_i + d_i\gamma + d_i\delta_i + 2\epsilon\gamma + \epsilon d_i + \epsilon\delta_i)\big] \end{aligned}$$

$$\begin{aligned} a_1 =& 2\epsilon\gamma^4 + (5\epsilon + 2\sigma)\gamma^3\delta_i + (2\epsilon\sigma + 2\sigma^2 + 2\sigma d_i)\gamma^3 + (2\epsilon\sigma + 2\sigma d_i + 2\sigma\delta_i + \epsilon d_i + 2\epsilon\delta_i)\gamma^2\delta_i \\ &+ (\sigma d_i + \sigma\delta_i + \epsilon\delta_i)\gamma\delta_i^2 + \sigma d_i\delta_i^2 + (\sigma^2 + \sigma d_i + \sigma\delta_i)\epsilon\gamma^2 + (\sigma^2 + \sigma d_i)\epsilon\gamma\delta_i \\ &+ \sigma^2\epsilon d_i\gamma + \sigma^2\epsilon d_i\delta_i + \epsilon\sigma d_i^2\delta_i \\ &- \sigma\lambda_{n,i}\big[(1-\pi)\rho(\epsilon\gamma^2 + \epsilon\gamma\delta_i + \epsilon d_i\gamma + \epsilon d_i\delta_i + 2\sigma\gamma^2 + \sigma\gamma\delta_i) \\ &+ (1-\pi)(1-\rho)(\epsilon\gamma^2 + \sigma\gamma^2 + \sigma\gamma\delta_i) + \iota\pi(\epsilon\gamma^2 + \sigma\gamma^2 + \sigma\gamma\delta_i)\big] \end{aligned}$$

$$\begin{aligned} a_0 =& \epsilon(\gamma + d_i)\big[\sigma\gamma(\delta_i + \gamma)^2 + \lambda_{n,i}\sigma(1-\pi)\rho(\delta_i + \gamma)^2 \\ &- \lambda_{n,i}\sigma(1-\pi)(1-\rho)\gamma(\delta_i + \gamma) + \iota\lambda_{n,i}\sigma\pi\gamma(\delta_i + \gamma)\big]. \end{aligned}$$

According to the Routh–Hurwitz condition, for $R_0 < 1$, the DFE is locally asymptotically stable if $a_6 > 0$, $a_5 > 0$, $a_4 > 0$, $a_3 > 0$, $a_2 > 0$, $a_1 > 0$, and $a_0 > 0$. That is,

$$a_6 = 1 > 0$$

$$a_5 = \sigma + 4\gamma + 2\delta_i + d_i + \epsilon > 0$$

$$\begin{aligned} a_4 = & 7\gamma^2 + 8\gamma\delta_i + 4\sigma\gamma + 3\sigma\delta_i + \delta_i^2 + 3d_i\delta_i + 3\epsilon\delta_i + \sigma d_i + 3\gamma d_i + \sigma\epsilon + 4\gamma\epsilon + 2d_i\epsilon \\ & - \lambda_{n,i}\sigma(1-\pi)\rho - \lambda_{n,i}\sigma(1-\pi)(1-\rho) - \iota\lambda_{n,i}\sigma\pi > 0 \end{aligned}$$

$$\begin{aligned} a_3 = & 4\gamma^3 + (6\epsilon + 6\sigma + 3d_i + \delta_i)\gamma^2 + 5\gamma^2\delta_i + (4\epsilon + 6\sigma + d_i)\gamma\delta_i + 2\gamma\delta_i^2 \\ & + (2\epsilon + 3\sigma + 2d_i + 2\delta_i)\sigma\gamma + (2\epsilon + 2\sigma + d_i)d_i\delta_i + (\epsilon + \sigma + d_i)\delta_i^2 + (\epsilon + \sigma)d_i\sigma + \epsilon\sigma\delta_i \\ & - \sigma\lambda_{n,i}\left[(1-\pi)\rho(\epsilon + 3\gamma + d_i + 2\delta_i) + (1-\pi)(1-\rho)(\epsilon + 3\gamma + d_i + \delta_i) + \iota\pi(\epsilon + 3\gamma + d_i + \delta_i)\right] > 0 \end{aligned}$$

$$\begin{aligned} a_2 = & 6\gamma^4 + 11\gamma^3\delta_i + (6\sigma + 3d_i + 2\epsilon)\gamma^3 + (8\sigma + 4d_i + 5\epsilon + 2\delta_i)\gamma^2\delta_i + (4\sigma + 2d_i + \epsilon + \delta_i)\gamma\delta_i^2 + (d_i + \epsilon)\delta_i^3 \\ & + (6\epsilon\sigma + 4\sigma^2 + 4\sigma d_i + 2\sigma\delta_i + 2\epsilon d_i + \epsilon\delta_i)\gamma^2 + (4\epsilon\sigma + 2\sigma^2 + 3\sigma d_i + 3\sigma\delta_i + \epsilon d_i)\gamma\delta_i \\ & + (\sigma^2 + \sigma d_i + \sigma\delta_i)d_i\delta_i + (\sigma d_i + \sigma\delta_i)\delta_i^2 + (\epsilon\sigma + \sigma^2)d_i^2 + \epsilon\sigma^2\gamma + \epsilon\sigma\delta_i^2 \\ & - \sigma\lambda_{n,i}\big[(1-\pi)\rho(5\gamma^2 + 5\gamma\delta_i + 2d_i\gamma + 2d_i\delta_i + 2\epsilon\gamma + \epsilon d_i + \epsilon\delta_i) \\ & + (1-\pi)(1-\rho)(3\gamma^2 + 2\gamma\delta_i + d_i\gamma + d_i\delta_i + 2\epsilon\gamma + \epsilon d_i + \epsilon\delta_i) \\ & + \iota\pi(3\gamma^2 + 2\gamma\delta_i + d_i\gamma + d_i\delta_i + 2\epsilon\gamma + \epsilon d_i + \epsilon\delta_i)\big] > 0 \end{aligned}$$

$$\begin{aligned} a_1 = & 2\epsilon\gamma^4 + (5\epsilon + 2\sigma)\gamma^3\delta_i + (2\epsilon\sigma + 2\sigma^2 + 2\sigma d_i)\gamma^3 + (2\epsilon\sigma + 2\sigma d_i + 2\sigma\delta_i + \epsilon d_i + 2\epsilon\delta_i)\gamma^2\delta_i \\ & + (\sigma d_i + \sigma\delta_i + \epsilon\delta_i)\gamma\delta_i^2 + \sigma d_i\delta_i^2 + (\sigma^2 + \sigma d_i + \sigma\delta_i)\epsilon\gamma^2 + (\sigma^2 + \sigma d_i)\epsilon\gamma\delta_i \\ & + \sigma^2\epsilon d_i\gamma + \sigma^2\epsilon d_i\delta_i + \epsilon\sigma d_i^2\delta_i \\ & - \sigma\lambda_{n,i}\big[(1-\pi)\rho(\epsilon\gamma^2 + \epsilon\gamma\delta_i + \epsilon d_i\gamma + \epsilon d_i\delta_i + 2\sigma\gamma^2 + \sigma\gamma\delta_i) \\ & + (1-\pi)(1-\rho)(\epsilon\gamma^2 + \sigma\gamma^2 + \sigma\gamma\delta_i) + \iota\pi(\epsilon\gamma^2 + \sigma\gamma^2 + \sigma\gamma\delta_i)\big] > 0 \end{aligned}$$

$$\begin{aligned} a_0 = & \epsilon(\gamma + d_i)\left[\sigma\gamma(\delta_i + \gamma)^2 + \lambda_{n,i}\sigma(1-\pi)\rho(\delta_i + \gamma)^2\right. \\ & \left. + \iota\lambda_{n,i}\sigma\pi\gamma(\delta_i + \gamma)\right] - \left[(\lambda_{n,i}\sigma(1-\pi)(1-\rho)\gamma(\delta_i + \gamma))\,\epsilon(\gamma + d_i)\right](R_0 - 1). \end{aligned}$$

Thus, $a_0 > 0$ if $R_0 < 1$. Since all the coefficients are positive, we conclude that for $R_0 < 1$, the DFE point of our model is locally stable. □

Theorem 4 (global stability)**.** *The system (1) is globally asymptotically stable at the DFE if $R_0 < 1$ and unstable if $R_0 > 1$.*

Proof. Let us consider the Lyapunov function V of the form

$$V(t, E, A, J, I) = aE_i^n + bA_i^n + cJ_i^n + dI_i^n. \tag{18}$$

Its derivative is

$$\begin{aligned} \frac{dV}{dt} = & \, a\Bigg[\sum_{k=1}^{n}\sum_{j=1}^{i}\boldsymbol{\lambda}_{i,n,j,k}\left(\mathbf{J}_{n,j} + \iota\mathbf{I}_{n,j} + \mathbf{A}_{n,j}\right)\frac{S_i^n}{N_i^n} - \sigma\left(((1-\pi)\rho + (1-\pi)(1-\rho) + \pi)\right)E_i^n\Bigg] \\ & + \, b\left[\sigma(1-\pi)\rho E_i^n - \gamma A_i^n\right] + \, c\left[\sigma(1-\pi)(1-\rho)E_i^n - (\delta_i + \gamma)J_i^n\right] + d\left[\sigma\pi E_i^n - (\delta_i + \gamma)I_i^n\right]. \end{aligned} \tag{19}$$

Rearranging such that like terms are collected together, and noting that at DFE, $S_i^n/N_i^n \leq 1$, we have

$$\begin{aligned} \frac{dV}{dt} \leq & \left[b\sigma(1-\pi)\rho + c\sigma(1-\pi)(1-\rho) + d\sigma\iota\pi - a\sigma\right]E_i^n + \left(a\sum_{k=1}^{n}\sum_{j=1}^{i}\boldsymbol{\lambda}_{i,n,j,k} - b\gamma\right)A_i^n \\ & + \left(a\sum_{k=1}^{n}\sum_{j=1}^{i}\boldsymbol{\lambda}_{i,n,j,k} - c(\delta_i + \gamma)\right)J_i^n + \left(a\sum_{k=1}^{n}\sum_{j=1}^{i}\boldsymbol{\lambda}_{i,n,j,k} - d(\delta_i + \gamma)\right)I_i^n. \end{aligned} \tag{20}$$

Let $a = 1/\sigma$, $b = \sum_{k=1}^{n}\sum_{j=1}^{i} \boldsymbol{\lambda}_{i,n,j,k}/\gamma\sigma$, $c = \sum_{k=1}^{n}\sum_{j=1}^{i} \boldsymbol{\lambda}_{n,k}/(\delta_i+\gamma)\sigma$ and $d = \iota \sum_{k=1}^{n}\sum_{j=1}^{i} \boldsymbol{\lambda}_{n,k}/(\delta_i+\gamma)\sigma$. Substituting these into equation (20) leads to

$$\frac{dV}{dt} \leq (R_0 - 1)E_i^n. \tag{21}$$

Hence, $\frac{dV}{dt} \leq (R_0 - 1)E_i^n$; it is important to note that $\frac{dV}{dt} = 0$ only at DFE. From LaSalle's invariance principle, it follows that all solutions of equation (1) converge to the DFE as $t \to \infty$ whenever $R_0 < 1$. The DFE is globally asymptotically stable when $R_0 < 1$. □

C.4 Existence of an Endemic Equilibrium Point

We calculate the endemic equilibrium by equating the system (1) to zero; then, we obtain $E_1 = (S_i^{n*}, E_i^{n*}, A_i^{n*}, J_i^{n*}, Q_i^{n*}, I_i^{n*}, H_i^{n*}, R_i^{n*}, D_i^{n*})$, such that

$$S_i^{n*} = \frac{\epsilon R_i^{n*}}{H^{**}} \tag{22a}$$

$$E_i^{n*} = \frac{H^{**} S_i^{n*}}{\sigma} \tag{22b}$$

$$A_i^{n*} = \frac{\sigma(1-\pi)\rho E_i^{n*}}{\gamma} \tag{22c}$$

$$J_i^{n*} = \frac{\sigma(1-\pi)\theta E_i^{n*}}{(\delta_i+\gamma)} \tag{22d}$$

$$I_i^{n*} = \frac{\sigma\pi E_i^{n*}}{(\delta_i+\gamma)} \tag{22e}$$

$$H_i^{n*} = \frac{\delta_i\sigma}{(\delta_i+d_i)(\delta_i+\gamma)}\left[(1-\pi)\theta+\pi\right]E_i^{n*} \tag{22f}$$

$$R_i^{n*} = \frac{\gamma}{\epsilon}\left[I_i^{n*} + A_i^{n*} + J_i^{n*} + H_i^{n*}\right]. \tag{22g}$$

From equation (7), we define

$$H^{**} = \sum_{k=1}^{n}\sum_{j=1}^{i} \frac{\boldsymbol{\lambda}_{i,n,j,k}}{N_i^n}\left(\mathbf{J}_{k,j} + \iota\mathbf{I}_{k,j} + \mathbf{A}_{k,j}\right). \tag{23}$$

At endemic equilibrium, we know that $H_i^{n*} \geq 0$ and that all our parameters are positive, so $d_i \neq 0$. We can conclude that D_i^{n*} depends on H_i^{n*}.

C.4.1 Uniqueness of the Endemic Equilibrium Point

Theorem 5. *An endemic equilibrium is unique iff $R_0 > 1$.*

Proof. If we rewrite equations (22c–22g) in terms of equation (22b), we have

$$A_i^{n*} = \frac{(1-\pi)\rho H^{**} S_i^{n*}}{\gamma} \tag{24a}$$

$$J_i^{n*} = \frac{(1-\pi)\theta H^{**} S_i^{n*}}{(\delta_i+\gamma)} \tag{24b}$$

$$I_i^{n*} = \frac{\pi H^{**} S_i^{n*}}{(\delta_i+\gamma)} \tag{24c}$$

$$H_i^{n*} = \frac{\delta_i}{(\delta_i+d_i)(\delta_i+\gamma)}\left[(1-\pi)\theta+\pi\right]H^{**} S_i^{n*}. \tag{24d}$$

Substituting A_i^{n*}, J_i^{n*}, I_i^{n*}, and H_i^{n*} from equations (24a–24d) into (22g), we obtain

$$R_i^{n*} = \frac{\gamma}{\epsilon}\left(\frac{(1-\pi)\rho H^{**}S_i^{n*}}{\gamma} + \frac{(1-\pi)\theta H^{**}S_i^{n*}}{(\delta_i+\gamma)} + \frac{\pi H^{**}S_i^{n*}}{(\delta_i+\gamma)} + \nu_1 H^{**}S_i^{n*}\right)\right) \tag{25}$$

where

$$\nu_1 = \frac{\delta_i}{(\delta_i+d_i)(\delta_i+\gamma)}\left[(1-\pi)\theta+\pi\right].$$

Equation (23) implies that

$$H^{**}N_i^{n*} = \sum_{k=1}^{n}\sum_{j=1}^{i}\boldsymbol{\lambda}_{i,n,j,k}\left(\mathbf{J}_{k,j}^{*} + \iota\mathbf{I}_{k,j}^{*} + \mathbf{A}_{k,j}^{*}\right), \tag{26}$$

which implies that

$$H^{**}P_2 = \sum_{k=1}^{n}\sum_{j=1}^{i}\boldsymbol{\lambda}_{i,n,j,k}\left(\frac{(1-\pi)\rho H^{**}S_i^{n*}}{\gamma} + \frac{(1-\pi)\theta H^{**}S_i^{n*}}{(\delta_i+\gamma)} + \iota\frac{\pi H^{**}S_i^{n*}}{(\delta_i+\gamma)}\right). \tag{27}$$

P_2 is derived from $N_i^{n*} = S_i^{n*} + E_i^{n*} + A_i^{n*} + J_i^{n*} + I_i^{n*} + H_i^{n*} + R_i^{n*} + D_i^{n*}$. Dividing both sides of equation (27) by $H^{**}S_i^{n*}$, we have

$$1 + P_3H^{**} = R_0. \tag{28}$$

Solving for H^{**} in equation (28), we have

$$H^{**} = \frac{R_0 - 1}{P_3} \tag{29}$$

and $P_3 > 0$. Here, P_3 is defined as

$$\frac{\mathbf{K} + \frac{1}{\sigma} + \nu_2}{\gamma\mathbf{K}},\ textWhere\,\mathbf{K} = \left(\frac{(1-\pi)\rho}{\gamma} + \frac{(1-\pi)\theta}{(\delta_i+\gamma)} + \frac{\pi}{(\delta_i+\gamma)} + \nu_1\right),\ \text{and}\ \nu_2 = \frac{\gamma}{\epsilon}\left(\frac{(1-\pi)\rho}{\gamma} + \frac{(1-\pi)\theta}{(\delta_i+\gamma)} + \frac{\pi}{(\delta_i+\gamma)} + \nu_1\right).$$

All parameters are positive; hence, $\gamma > 0$ and $\mathbf{K} > 0$, so $\gamma\mathbf{K} > 0$. Therefore, $H^{**} > 0$ iff $R_0 > 1$. Therefore, a unique endemic equilibrium exists. □

C.5 Global Stability of the Endemic Equilibrium

Theorem 6 (global stability of endemic equilibrium)**.** *The endemic equilibrium E_1 of the model system (1) is globally asymptotically stable if $R_0 > 1$.*

Proof. To prove global stability, we used the Lyapunov function method. Consider the Lyapunov function of the form

$$L = \left(S_i^n - S_i^{n*} - S_i^{n*}\log\frac{S_i^n}{S_i^{n*}}\right) + \left(E_i^n - E_i^{n*} - E_i^{n*}\log\frac{E_i^n}{E_i^{n*}}\right) + \left(A_i^n - A_i^{n*} - A_i^{n*}\log\frac{A_i^n}{A_i^{n*}}\right) + \left(J_i^n - J_i^{n*} - J_i^{n*}\log\frac{J_i^n}{J_i^{n*}}\right)$$
$$+ \left(I_i^n - I_i^{n*} - I_i^{n*}\log\frac{I_i^n}{I_i^{n*}}\right) + \left(H_i^n - H_i^{n*} - H_i^{n*}\log\frac{H_i^n}{H_i^{n*}}\right) + \left(R_i^n - R_i^{n*} - R_i^{n*}\log\frac{R_i^n}{R_i^{n*}}\right) + \left(D_i^n - D_i^{n*} - D_i^{n*}\log\frac{D_i^n}{D_i^{n*}}\right).$$

Taking the derivative of L, we obtain

$$\frac{dL}{dt} = \left(\frac{S_i^n - S_i^{n*}}{S_i^n}\right)\frac{dS_i^n}{dt} + \left(\frac{E_i^n - E_i^{n*}}{E_i^n}\right)\frac{dE_i^n}{dt} + \left(\frac{A_i^n - A_i^{n*}}{A_i^n}\right)\frac{dA_i^n}{dt} + \left(\frac{J_i^n - J_i^{n*}}{J_i^n}\right)\frac{dJ_i^n}{dt} + \left(\frac{I_i^n - I_i^{n*}}{I_i^n}\right)\frac{dI_i^n}{dt}$$
$$+ \left(\frac{H_i^n - H_i^{n*}}{H_i^n}\right)\frac{dH_i^n}{dt} + \left(\frac{R_i^n - R_i^{n*}}{R_i^n}\right)\frac{dR_i^n}{dt} + \left(\frac{D_i^n - D_i^{n*}}{D_i^n}\right)\frac{dD_i^n}{dt}. \tag{30}$$

Substituting the value of the derivatives into (30) yields

$$
\begin{aligned}
\frac{dL}{dt} = &\left(\frac{S_i^n - S_i^{n*}}{S_i^n}\right)\left[-(\boldsymbol{\beta})_{n,i}(t)\frac{S_i^n}{N_i^n} + \epsilon R_i^n\right] + \left(\frac{E_i^n - E_i^{n*}}{E_i^n}\right)\left[(\boldsymbol{\beta})_{n,i}(t)\frac{S_i^n}{N_i^n} - \sigma E_i^n\right] + \left(\frac{A_i^n - A_i^{n*}}{A_i^n}\right)[\sigma\,(1-\pi(t))\,\rho E_i^n - \gamma A_i^n] \\
&+ \left(\frac{J_i^n - J_i^{n*}}{J_i^n}\right)[\sigma(1-\pi(t))\theta E_i^n - \delta_i J_i^n - \gamma J_i^n] + \left(\frac{I_i^n - I_i^{n*}}{I_i^n}\right)[\sigma\pi E_i^n - \delta_i I_i^n - \gamma I_i^n] \\
&+ \left(\frac{H_i^n - H_i^{n*}}{H_i^n}\right)[\delta_i J_i^n + \delta_i I_i^n - \gamma H_i^n - d_i H_i^n] + \left(\frac{R_i^n - R_i^{n*}}{R_i^n}\right)[(I_i^n + A_i^n + J_i^n + H_i^n)\,\gamma - \epsilon R_i^n] \\
&+ \left(\frac{D_i^n - D_i^{n*}}{D_i^n}\right) d_i H_i^n.
\end{aligned}
$$

By substituting $(\beta)_{n,i}$, we obtain

$$
\begin{aligned}
\frac{dL}{dt} = &\frac{S_i^n - S_i^{n*}}{S_i^n}\left[-\frac{\sum_{k=1}^{n}\sum_{j=1}^{i}\boldsymbol{\lambda}_{i,n,j,k}}{N_i^n}\Big((J_i^n - J_i^{n*})(S_i^n - S_i^{n*}) + \iota\,(I_i^n - I_i^{n*})(S_i^n - S_i^{n*}) + (A_i^n - A_i^{n*})(S_i^n - S_i^{n*})\Big) + \epsilon\,(R_i^n - R_i^{n*})\right] \\
&+ \frac{E_i^n - E_i^{n*}}{E_i^n}\left[\frac{\sum_{k=1}^{n}\sum_{j=1}^{i}\boldsymbol{\lambda}_{i,n,j,k}}{N_i^n}\Big((J_i^n - J_i^{n*})(S_i^n - S_i^{n*}) + \iota\,(I_i^n - I_i^{n*})(S_i^n - S_i^{n*}) + (A_i^n - A_i^{n*})(S_i^n - S_i^{n*})\Big) - (E_i^n - E_i^n)\right] \\
&+ \frac{A_i^n - A_i^{n*}}{A_i^n}\left[\sigma\,(1-\pi(t))\,\rho\,E_i^n \;-\; \gamma\,A_i^n\right] \\
&+ \frac{J_i^n - J_i^{n*}}{J_i^n}\left[\sigma\,(1-\pi(t))\,\theta\,E_i^n \;-\; (\delta_i + \gamma)\,J_i^n\right] \\
&+ \frac{I_i^n - I_i^{n*}}{I_i^n}\left[\sigma\,\pi\,E_i^n \;-\; (\delta_i + \gamma)\,I_i^n\right] \\
&+ \frac{H_i^n - H_i^{n*}}{H_i^n}\left[\delta_i\,(J_i^n + I_i^n) \;-\; (\gamma + d_i)\,H_i^n\right] \\
&+ \frac{R_i^n - R_i^{n*}}{R_i^n}\left[\gamma\,(I_i^n + A_i^n + J_i^n + H_i^n) \;-\; \epsilon\,R_i^n\right] \\
&+ \frac{D_i^n - D_i^{n*}}{D_i^n}\;d_i\,H_i^n\,. \qquad (31)
\end{aligned}
$$

After some algebra, it is possible to split equation (31) into positive and negative parts:

$$
\begin{aligned}
\frac{dL^{+}}{dt} = {} & \frac{S_i^n - S_i^{n*}}{S_i^n}\,\epsilon\,(R_i^n - R_i^{n*}) \\
& + \frac{E_i^n - E_i^{n*}}{E_i^n}\,\frac{\sum_{k=1}^{n}\sum_{j=1}^{i}\boldsymbol{\lambda}_{i,n,j,k}}{N_i^n}\Big[(J_i^n - J_i^{n*})(S_i^n - S_i^{n*}) + \iota\,(I_i^n - I_i^{n*})(S_i^n - S_i^{n*}) + (A_i^n - A_i^{n*})(S_i^n - S_i^{n*})\Big] \\
& + \frac{A_i^n - A_i^{n*}}{A_i^n}\big[\sigma(1-\pi(t))\,\rho\,E_i^n \;-\; \gamma\,A_i^n\big] \\
& + \frac{J_i^n - J_i^{n*}}{J_i^n}\big[\sigma(1-\pi(t))\,\theta\,E_i^n \;-\; (\delta_i + \gamma)\,J_i^n\big] \\
& + \frac{I_i^n - I_i^{n*}}{I_i^n}\big[\sigma\,\pi\,E_i^n \;-\; (\delta_i + \gamma)\,I_i^n\big] \\
& + \frac{H_i^n - H_i^{n*}}{H_i^n}\big[\delta_i\,(J_i^n + I_i^n) \;-\; (\gamma + d_i)\,H_i^n\big] \\
& + \frac{R_i^n - R_i^{n*}}{R_i^n}\big[\gamma\,(I_i^n + A_i^n + J_i^n + H_i^n) \;-\; \epsilon\,R_i^n\big] \\
& + \frac{D_i^n - D_i^{n*}}{D_i^n}\,d_i\,H_i^n\,.
\end{aligned} \tag{32}
$$

Taking the positive terms, we have

$$
\begin{aligned}
\frac{dL^{+}}{dt} = {} & \frac{(S_i^n - S_i^{n*})^2}{S_i^n}\,\frac{\sum_{k=1}^{n}\sum_{j=1}^{i}\boldsymbol{\lambda}_{i,n,j,k}}{N_i^n}\,[J_i^{n*} + I_i^{n*} + A_i^{n*}] + \frac{(S_i^n - S_i^{n*})}{S_i^n}\epsilon R_i^n \\
& + \frac{(E_i^n - E_i^{n*})}{E_i^n}\,\frac{\sum_{k=1}^{n}\sum_{j=1}^{i}\boldsymbol{\lambda}_{i,n,j,k}}{N_i^n}\,[I_i^n S_i^n + I_i^{n*} S_i^{n*} + A_i^n S_i^n + A_i^{n*} S_i^{n*} + J_i^n S_i^n + J_i^{n*} S_i^{n*}] + \frac{(A_i^n - A_i^{n*})}{A_i^n}\,[\sigma\rho E_i^n + \sigma\pi\rho E_i^{n*}] \\
& + \frac{(J_i^n - J_i^{n*})}{J_i^n}\,[\sigma\theta E_i^n + \sigma\pi\theta E_i^{n*}] + \frac{(I_i^n - I_i^{n*})}{I_i^n}\,[\sigma\pi E_i^n] + \frac{(H_i^n - H_i^{n*})}{H_i^n}\,[\delta_i I_i^n + \delta_i J_i^n] \\
& + \frac{(R_i^n - R_i^{n*})}{R_i^n}\,[\gamma I_i^n + \gamma A_i^n + \gamma J_i^n + \gamma H_i^n] + \frac{(D_i^n - D_i^{n*})}{D_i^n} d_i H_i^n.
\end{aligned} \tag{33}
$$

Taking the negative terms, we have

$$
\begin{aligned}
\frac{dL^{-}}{dt} = {} & -\left(\frac{(S_i^n - S_i^{n*})^2}{S_i^n}\,\frac{\sum_{k=1}^{n}\sum_{j=1}^{i}\boldsymbol{\lambda}_{i,n,j,k}}{N_i^n}\,[J_i^n + I_i^n + A_i^n]\right) - \frac{(S_i^n - S_i^{n*})}{S_i^n}\epsilon R_i^{n*} \\
& - \frac{(E_i^n - E_i^{n*})^2}{E_i^n}\sigma\left(\frac{E_i^n - E_i^{n*}}{E_i^n}\right)\frac{\sum_{k=1}^{n}\sum_{j=1}^{i}\boldsymbol{\lambda}_{i,n,j,k}}{N_i^n}\,[J_i^n S_i^{n*} + J_i^{n*} S_i^n + I_i^n S_i^{n*} + I_i^{n*} S_i^n + A_i^n S_i^{n*} + A_i^{n*} S_i^n] \\
& - \left(\frac{A_i^n - A_i^{n*}}{A_i^n}\right)[\sigma\pi\rho E_i^n + \sigma\pi\rho E_i^{n*}] - \left(\frac{J_i^n - J_i^{n*}}{J_i^n}\right)[\sigma E_i^{n*} + \sigma\rho E_i^n + \sigma\pi E_i^n \sigma\pi E_i^{n*}] - \left(\frac{I_i^n - I_i^{n*}}{I_i^n}\right)[\sigma\pi E_i^{n*}] \\
& - \left(\frac{H_i^n - H_i^{n*}}{H_i^n}\right)[\delta_i I_i^{n*} + \delta_i J_i^{n*}]\left(\frac{R_i^n - R_i^{n*}}{R_i^n}\right)[\gamma I_i^{n*} + \gamma J_i^{n*} + \gamma A_i^{n*} + \gamma H_i^{n*}] - \frac{(D_i^n - D_i^{n*})}{D_i^n} d_i H_i^{n*}.
\end{aligned} \tag{34}
$$

We can write $\frac{dL}{dt} = \mathbf{A} - \mathbf{B}$, where $\mathbf{A}$ represents the positive terms (33) and $\mathbf{B}$ the negative terms (34). Thus, if $\mathbf{A} < \mathbf{B}$, then we obtain that $\frac{dL}{dt} \leq 0$ iff $(S_i^n = S_i^{n*}, E_i^n = E_i^{n*}, A_i^n = A_i^{n*}, J_i^n = J_i^{n*}, I_i^n = I_i^{n*}, H_i^n = H_i^{n*}, R_i^n = R_i^{n*}, D_i^n = D_i^{n*})$. Therefore, the largest compact invariant set in $(S_i^{n*}, E_i^{n*}, A_i^{n*}, J_i^{n*}, I_i^{n*}, H_i^{n*}, R_i^{n*}, D_i^{n*}) \in \Omega : \frac{dL}{dt} = 0$ is the singleton set, E_{eff}, where E_{eff} is the endemic equilibrium of the system. According to LaSalle's invariance principle, this implies that E_1 is globally asymptotically stable in Ω if $\mathbf{A} < \mathbf{B}$. □

Appendix D Sensitivity Analysis

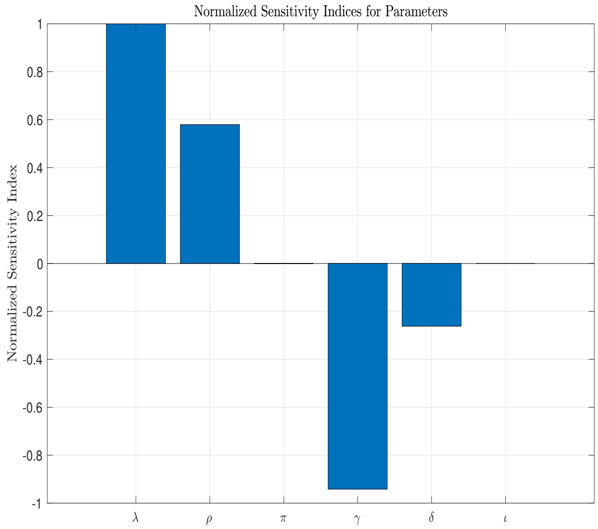

Fig 3. Bar chart of normalised sensitivity indices for key model parameters affecting the basic reproduction number R_0. Each bar represents the relative impact of changes in the model parameters, with positive values indicating parameters that increase R_0 and negative values indicating those that decrease it. High sensitivity values imply that small changes in these parameters can significantly affect the disease transmission. Key parameters include the contact rate λ, recovery rate γ, and hospitalisation rate δ.

We performed a sensitivity analysis to study how the uncertainty in the model output could be predicted by the uncertainty in the model input. This analysis helps the mathematical modeller validate their assumptions and improve trust in the results of the model. The normalised sensitivity index for each parameter X within R_0 is defined as

$$S_X^{R_0} = \frac{\partial R_0}{\partial X} \times \frac{X}{R_0},$$

which was used in studies by Wodajo *et al.* [6] and Al-arydah [7].

Parameters with a high sensitivity index have a greater influence on the R_0 value. The sign of the index indicates whether the parameter influences R_0 positively or negatively. From Figure 3, it can be seen that the contact rate, proportion of asymptomatic individuals in the population, testing rate, recovery rate, and rate of hospitalisation are the key parameters that influence the reproduction number, whereas the sensitivity of the test and average number of days for the test to be reported have a small influence.

The sensitivity indices were expressed as follows:

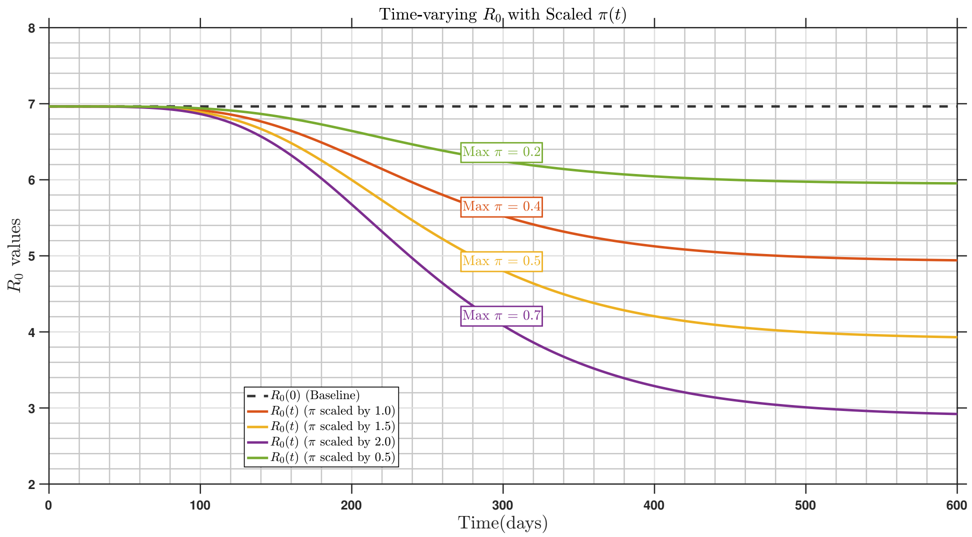

Fig 4. Time-varying basic reproduction number $R_0(t)$ under different testing rate trajectories $\pi(t)$. The dashed horizontal line marks the initial reproduction number $R_0(0) \approx 7.0$. Solid curves show how $R_0(t)$ declines as the cumulative testing capacity increases according to a logistic "generalized Richard" curve scaled by factors 0.5, 1.0, 1.5, and 2.0 (yielding maximum proportions of the testpopulation of 0.2, 0.4, 0.5, and 0.7, respectively). Increased testing reduces the proportion of undetected (and thus fully transmissible) infections, lowering R_0, but—even at the highest feasible testing scale—cannot on its own drive R_0 below 1.

1. The sensitivity index for $\lambda_{i,n,j,k}$ is

$$\sum_{k=1}^{10}\sum_{j=1}^{21} S_{\lambda_{i,n,j,k}} = \frac{1}{R_0}\sum_{k=1}^{10}\sum_{j=1}^{21} \lambda_{i,n,j,k}\, A_j = \frac{R_0}{R_0} = 1\,,$$

   where $A_j = \frac{(1-\pi)\rho}{\gamma} + \frac{(1-\pi)(1-\rho)}{\delta_j+\gamma} + \frac{\iota\pi}{\delta_j+\gamma}$. This indicates that the normalised sensitivity index of $\lambda_{i,n,j,k}$ is a matrix of ones.

2. The sensitivity index for γ is

$$S_\gamma = \frac{1}{R_0}\sum_{k=1}^{10}\sum_{j=1}^{21} \lambda_{i,n,j,k}\left[-\frac{(1-\pi)\rho}{\gamma} - \frac{\gamma\left((1-\pi)(1-\rho)+\iota\pi\right)}{(\delta_j+\gamma)^2}\right]. \qquad (35)$$

3. The sensitivity index for ρ is

$$S_\rho = \frac{\rho}{R_0}\sum_{k=1}^{10}\sum_{j=1}^{21} \lambda_{i,n,j,k}\left[\frac{1-\pi}{\gamma} - \frac{1-\pi}{\delta_j+\gamma}\right].$$

4. The sensitivity index for δ_j is

$$S_{\delta_j} = \frac{\delta_j}{R_0}\sum_{k=1}^{10}\sum_{j=1}^{21} \lambda_{i,n,j,k}\left[-\frac{(1-\pi)(1-\rho)+\iota\pi}{(\delta_j+\gamma)^2}\right].$$

However, we can compute the sensitivity index specific to each j by summing only over the index k. The normalised sensitivity index for δ_j is given by

$$S_{\delta_j} = \frac{\delta_j}{R_0} \sum_{k=1}^{10} \lambda_{i,n,j,k} \left[-\frac{(1-\pi)(1-\rho)+\iota\pi}{(\delta_j+\gamma)^2} \right].$$

5. The sensitivity index for π is

$$S_{\pi} = \frac{\pi}{R_0} \left(\sum_{k=1}^{10} \sum_{j=1}^{21} \lambda_{i,n,j,k} \left[-\frac{\rho}{\gamma} - \frac{1-\rho}{\delta_j+\gamma} + \frac{\iota}{\delta_j+\gamma} \right] \right). \tag{36}$$

6. The sensitivity index for ι is

$$S_{\iota} = \frac{\iota}{R_0} \sum_{k=1}^{10} \sum_{j=1}^{21} \lambda_{i,n,j,k} \frac{\pi}{\delta_j+\gamma}.$$

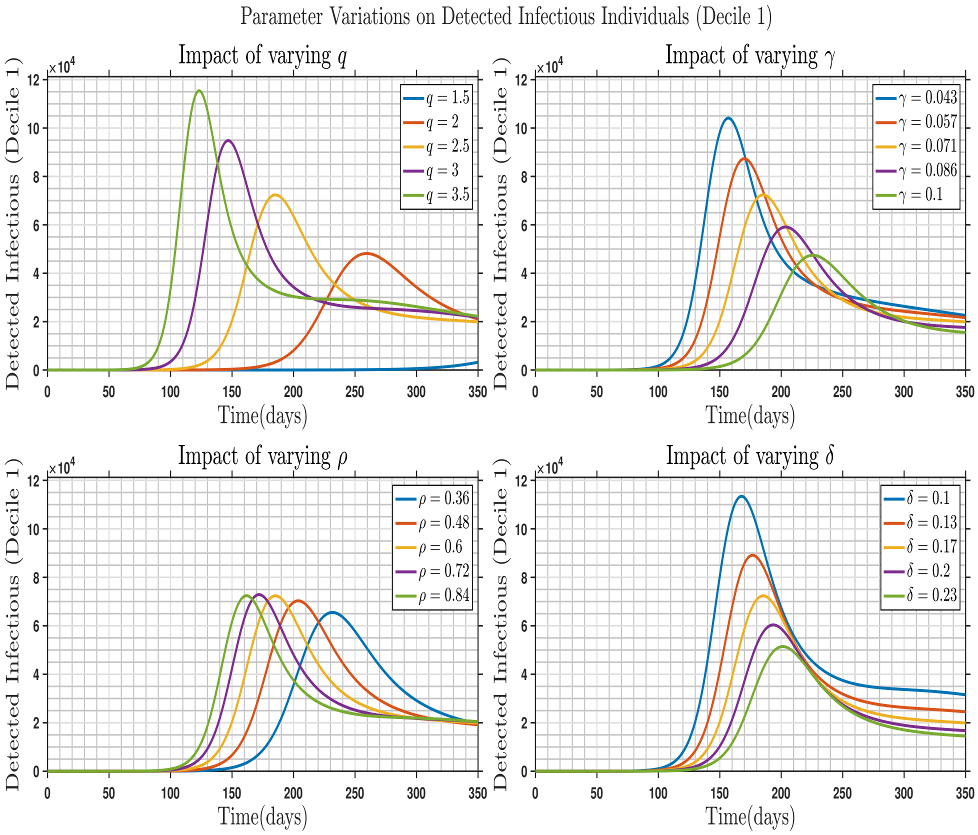

Fig 5. Sensitivity of infection dynamics in the most-deprived decile (Decile 1) when $R_0 > 1$. Each panel shows the time series of detected infectious individuals under $\pm 20\%$ variation from baseline values in some key parameters: relative transmission rate $\hat{q}$, proportion of asymptomatic ρ, recovery rate γ, and the rate of hospitalisation δ. Changes in these parameters can have a significant impact on the peak of infection.

Appendix E Numerical Analysis

We numerically analysed the sensitivity indices associated with each parameter. Numerical analysis was performed in MATLAB using the built-in ode45 solver with the parameter values listed in Table 1. The initial conditions for each age group and deprivation decile were set such that the entire population was susceptible, except for a small seed of latent infections ($E_{15}^{1}(0) = 1$) to initiate spread. The parameter values were obtained from existing literature, except where otherwise stated. A sensitivity analysis was performed using the sensitivity index method and numerically by perturbing some of the parameters to observe their impact on the system dynamics, as presented in Figure 5.

E.1 Simulation of model (1) at $R_0 > 1$

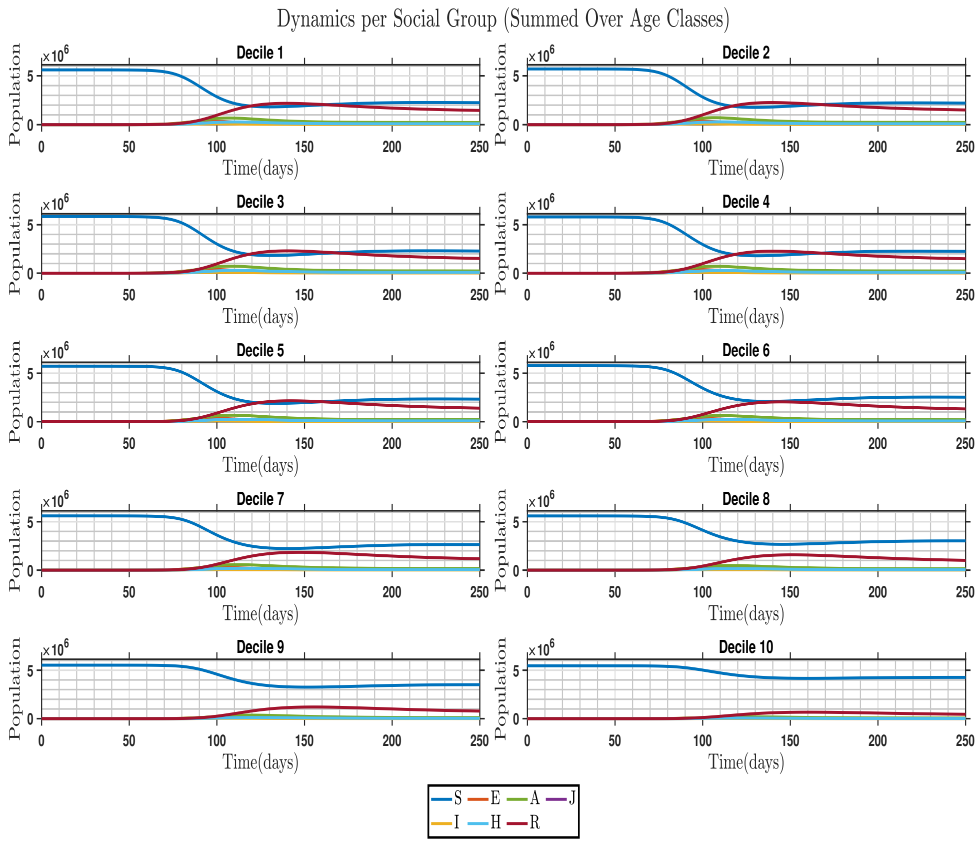

Fig 6. Time series of all epidemiological compartments aggregated over age for each deprivation decile. Panels1–10 correspond to deciles1 (most deprived) to10 (least deprived). For each, the curves show the susceptible, exposed, asymptomatic, symptomatic (detected and undetected), hospitalised, and recovered populations over 150 days. More deprived deciles peak earlier and at higher magnitudes in infectious and hospitalised cases, reflecting denser contact patterns.

In Figure 6, we plotted the full time series of the model's eight compartments (susceptible, exposed, asymptomatic, undetected, and detected symptomatic, hospitalised, recovered, and deceased) aggregated over age for each deprivation decile using the baseline parameter set (Table 1). Deciles1–10 are shown in separate panels, highlighting that the most-deprived groups experience earlier and larger infection peaks.

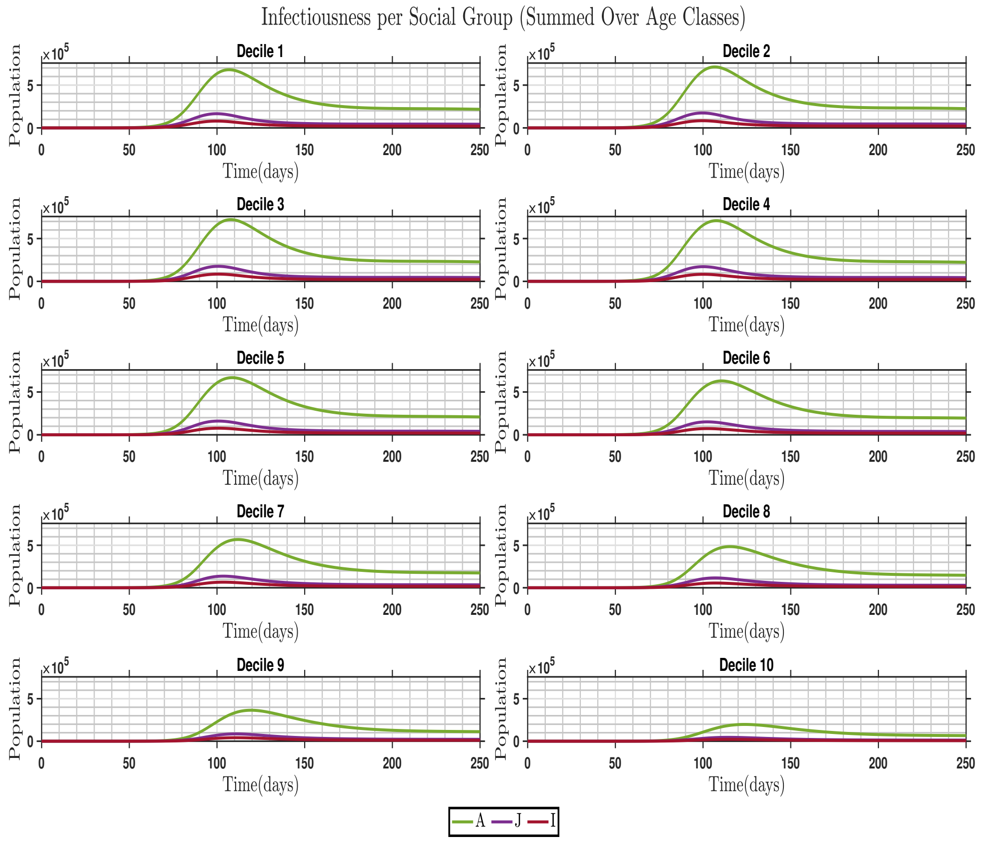

Fig 7. Infection trajectories by deprivation decile, aggregated over age groups. For each decile (1=most deprived. . . 10=least deprived), the curves show asymptomatic (green), detected symptomatic (red), and undetected symptomatic (orange), populations over 250 days. More-deprived deciles peak earlier and reach higher burdens in the infectious compartments.

In Figure 7, we focus on the breakdown of the infectious compartments, again summed over age within each decile, and using the baseline parameter set (Table 1).

E.2 Pairwise variation of key epidemiological parameters

In Figure 8, we hold the age–deprivation contact matrix fixed and compute R_0 across a $\pm20\%$ grid of the relative transmission rate $\hat{q}$, proportion of asymptomatic individuals ρ, recovery rate γ, and hospitalisation rate δ. Darker shading indicates a higher R_0. We see that R_0 is most sensitive to $\hat{q}$ and γ; reducing transmission or shortening the infectious duration yields the greatest decline in R_0.

Figure 9 repeats these comparisons but now varies the overall contact-mixing scale λ vs. each of ρ, γ, and δ in the same six-panel layout. We deduce that interventions aimed at minimising contact, in conjunction with key measures such as improving recovery through treatment, are crucial to effectively manage the spread of the disease.

References

1. Mossong J, et al. Social contacts and mixing patterns relevant to the spread of infectious diseases. PLoS Medicine. 2008;5:e74. doi:10.1371/journal.pmed.0050074.

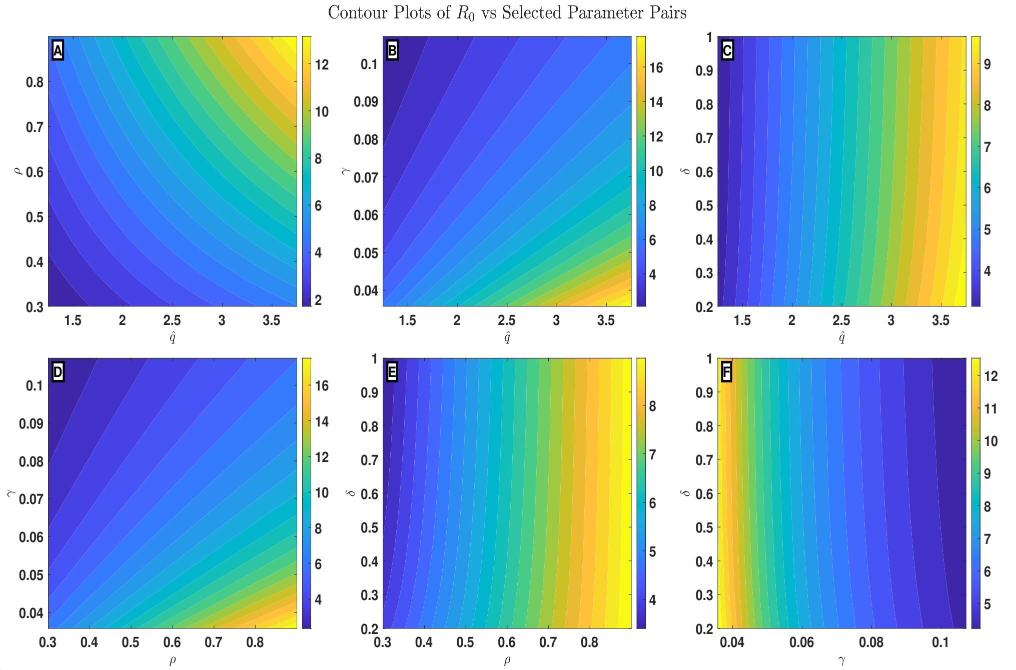

Fig 8. Contour plots illustrating the sensitivity of the basic reproduction number R_0 to pairwise variations in the key epidemiological parameters. Each panel shows R_0 computed over a $\pm 20\,\%$ grid around the baseline values of the two parameters, with all other parameters fixed. δ_i the rate of hospitalisation by age group is scaled to δ by the value $[0.2, 1]$. The pairs of parameters are (A) relative transmission rate $\hat{q}$ vs asymptomatic fraction ρ, (B) $\hat{q}$ vs recovery rate γ, (C) $\hat{q}$ vs hospitalisation rate δ, (D) ρ vs γ, (E) ρ vs δ, and (F) γ vs. δ. Shading denotes the magnitude of R_0 according to the adjacent colour bar.

2. Hethcote HW. Modeling heterogeneous mixing in infectious disease dynamics. In: Models for infectious human diseases: their structure and relation to data. vol. 215; 1996. p. 238.

3. Hale AC, Read JM, Jewell C. Modelling the impact of social mixing and behaviour on infectious disease transmission: application to SARS-CoV-2; 2022. Available from: `https://doi.org/10.48550/arXiv.2211.02371`.

4. Wu K, Darcet D, Wang Q, Sornette D. Generalized logistic growth modeling of the COVID-19 outbreak: comparing the dynamics in the 29 provinces in China and in the rest of the world. Nonlinear Dynamics. 2020;101:1561–1581. doi:10.1007/s11071-020-05862-6.

5. UK Government. Coronavirus (COVID-19) in the UK; 2023. Available from: `https://coronavirus.data.gov.uk/details/testing?areaType=nation&areaName=England`.

6. Wodajo FA, Gebru DM, Alemneh HT. Mathematical model analysis of effective intervention strategies on transmission dynamics of hepatitis B virus. Scientific Reports. 2023;13:8737. doi:10.1038/s41598-023-35967-7.

7. Al-Arydah M. Assessing vaccine efficacy for infectious diseases with variable immunity using a mathematical model. Scientific Reports. 2024;14:18572. doi:10.1038/s41598-024-69266-2.

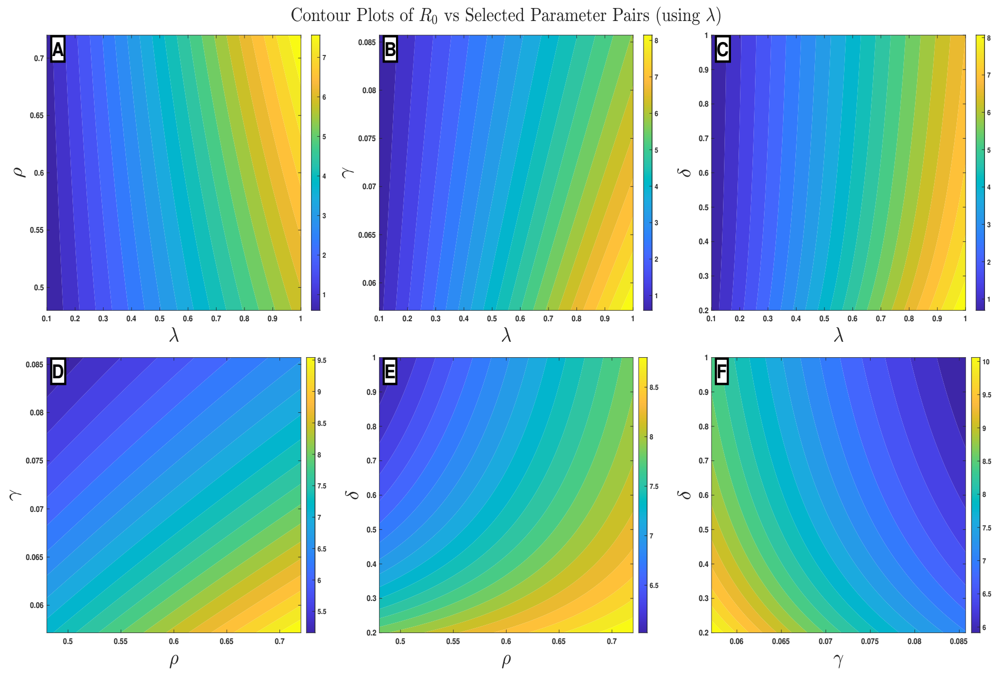

Fig 9. Contour plots illustrating the sensitivity of the basic reproduction number R_0 to pairwise variations in key epidemiological parameters. Each panel shows R_0 computed over a $\pm 20\,\%$ grid around the baseline values of the two parameters, with all other parameters fixed. The contact mixing matrix is scaled between $[0.1, 1]$ and δ_i the rate of hospitalisation by age group is scaled to δ by the value $[0.2, 1]$. The pairs of parameters are (A) scaled contact matrix λ vs asymptomatic fraction ρ, (B) λ vs recovery rate γ, (C) λ vs hospitalisation rate δ, (D) ρ vs γ, (E) ρ vs δ, and (F) γ vs δ. The shading denotes the magnitude of R_0 according to the adjacent colour bar.
